# Supplementary material for: Genome sequencing and molecular networking analysis of the wild fungus Anthostomella pinea reveal its ability to produce a diverse range of secondary metabolites
Source: Fungal Biol Biotechnol. 2024 Jan 3;11:1. doi: 10.1186/s40694-023-00170-1 (PMC10763133; doi:10.1186/s40694-023-00170-1)

GNPS hit 1 (MEB) - XIC range m/z 233.152-233.154 [M+H]

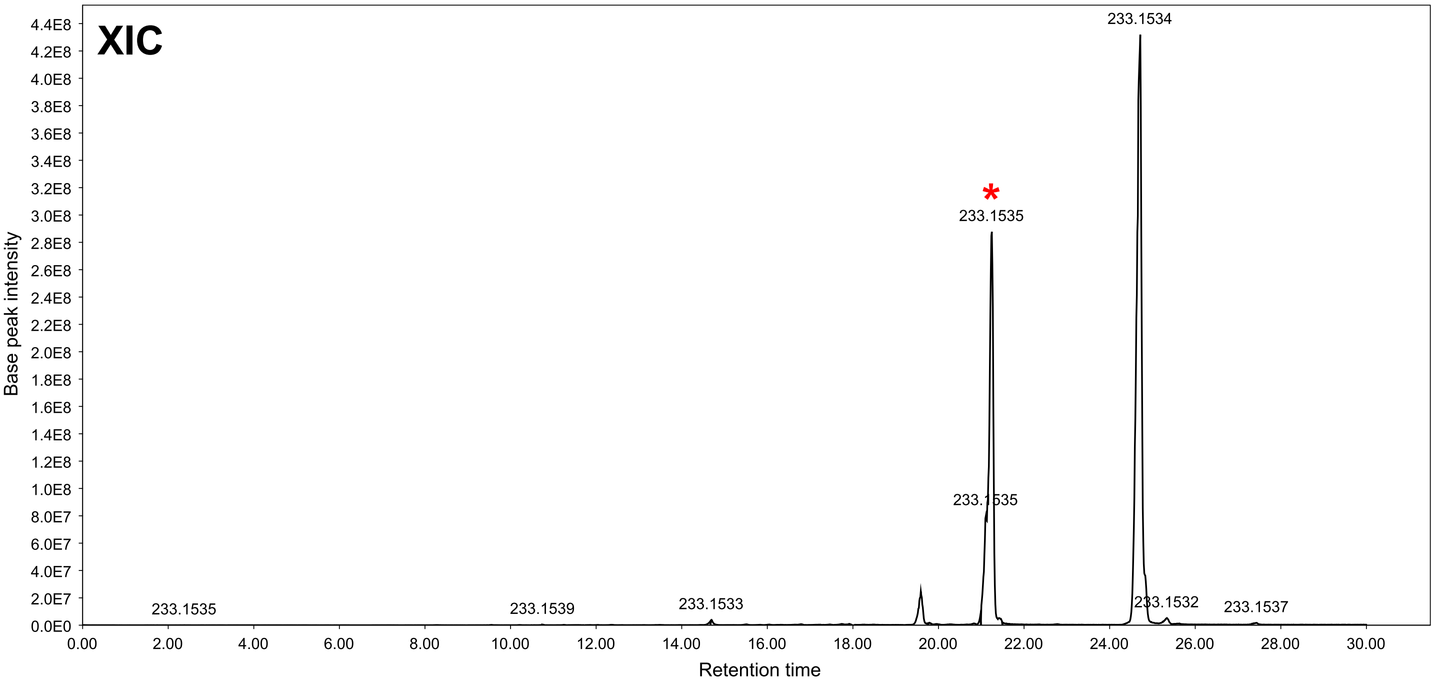

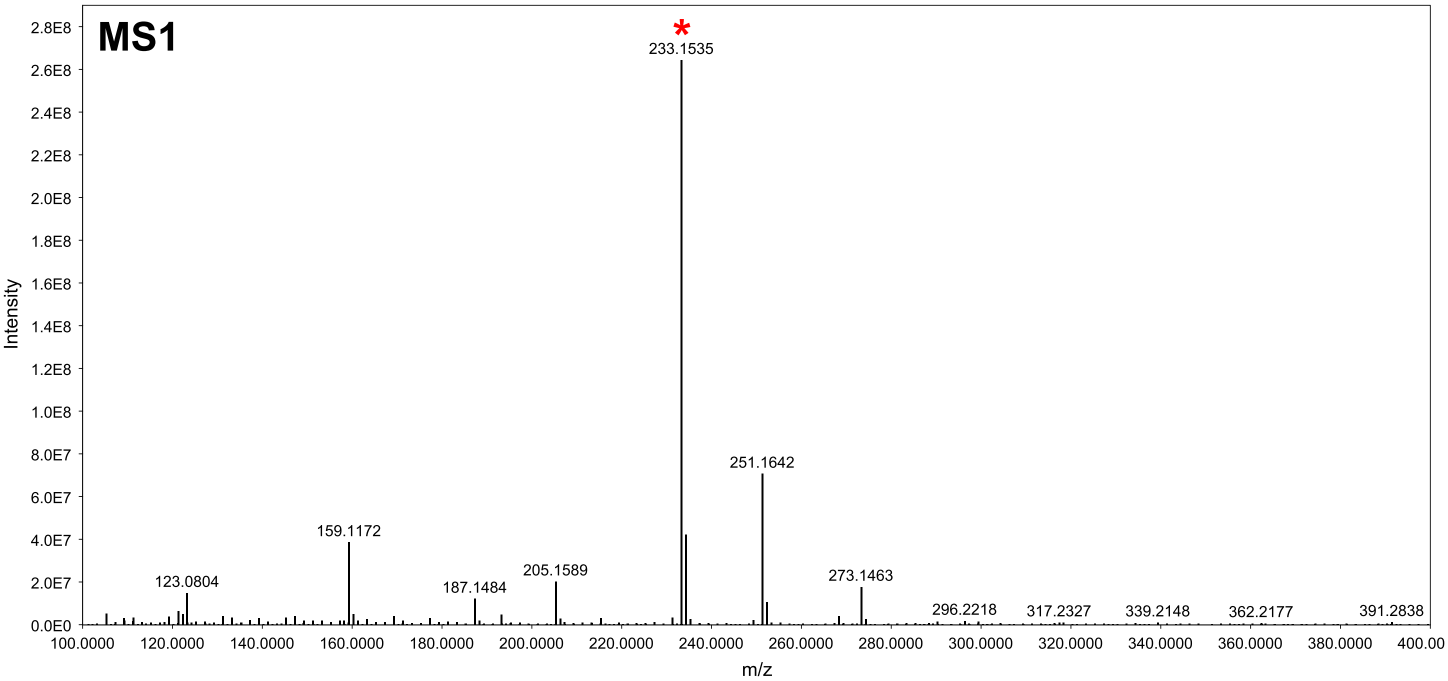

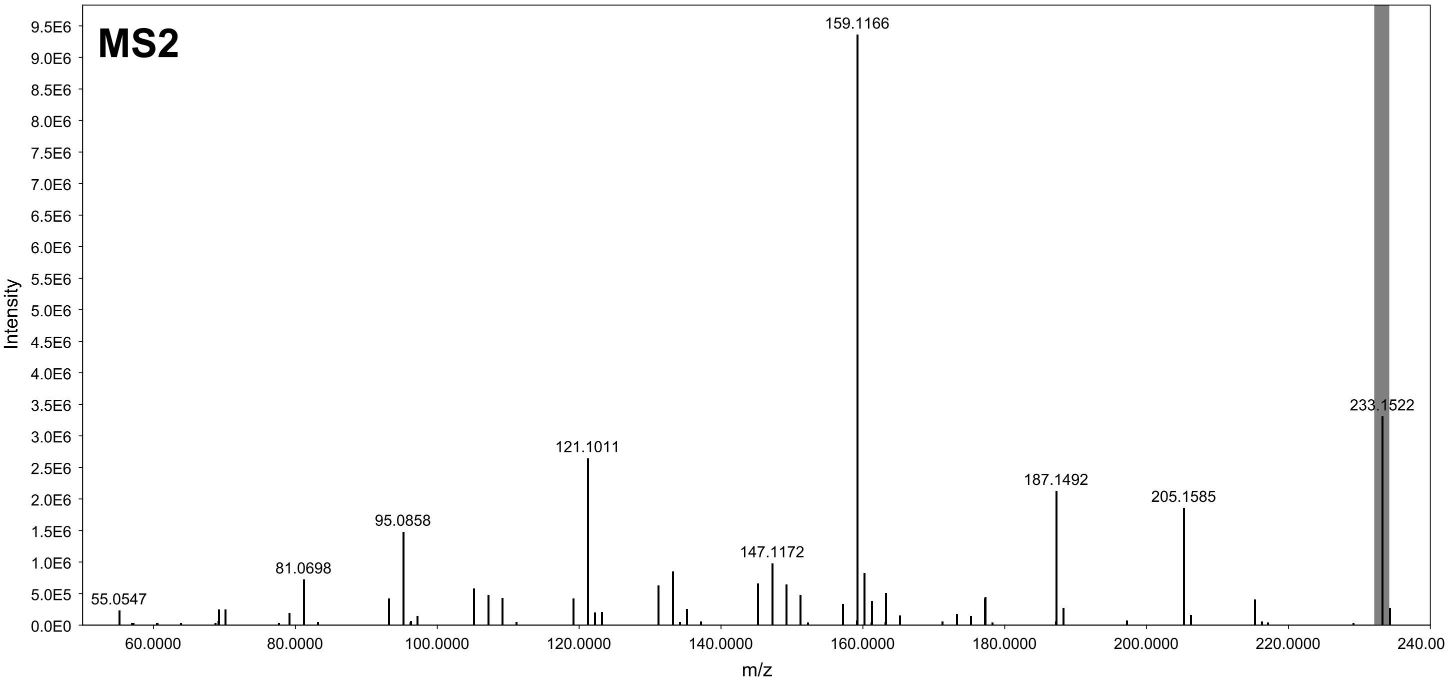

GNPS hit 2 (MEB) - XIC range m/z 251.199-251.201 [M+H]

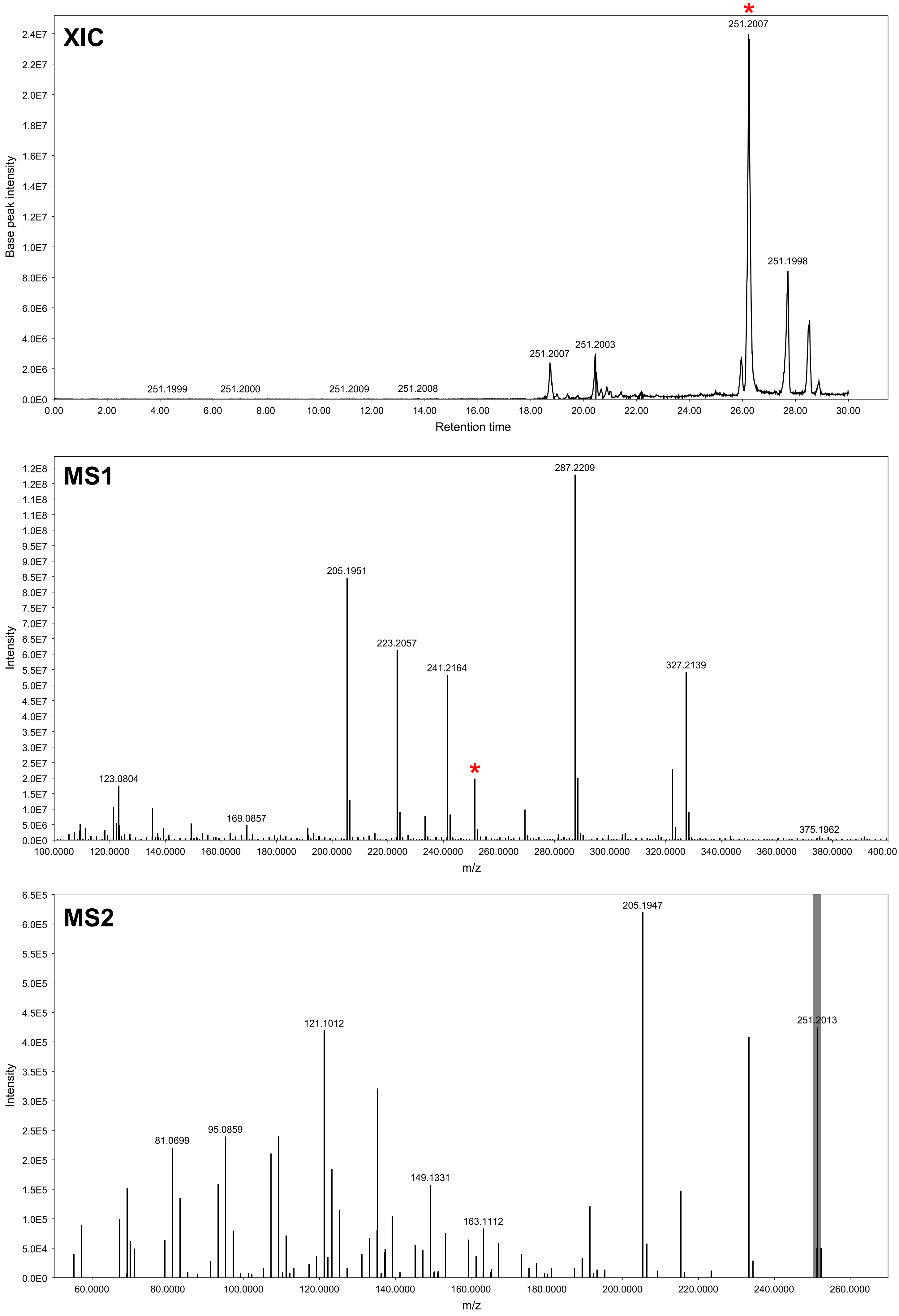

GNPS hit 3 (MEB) - XIC range m/z 223.204-22.206 [M+H]

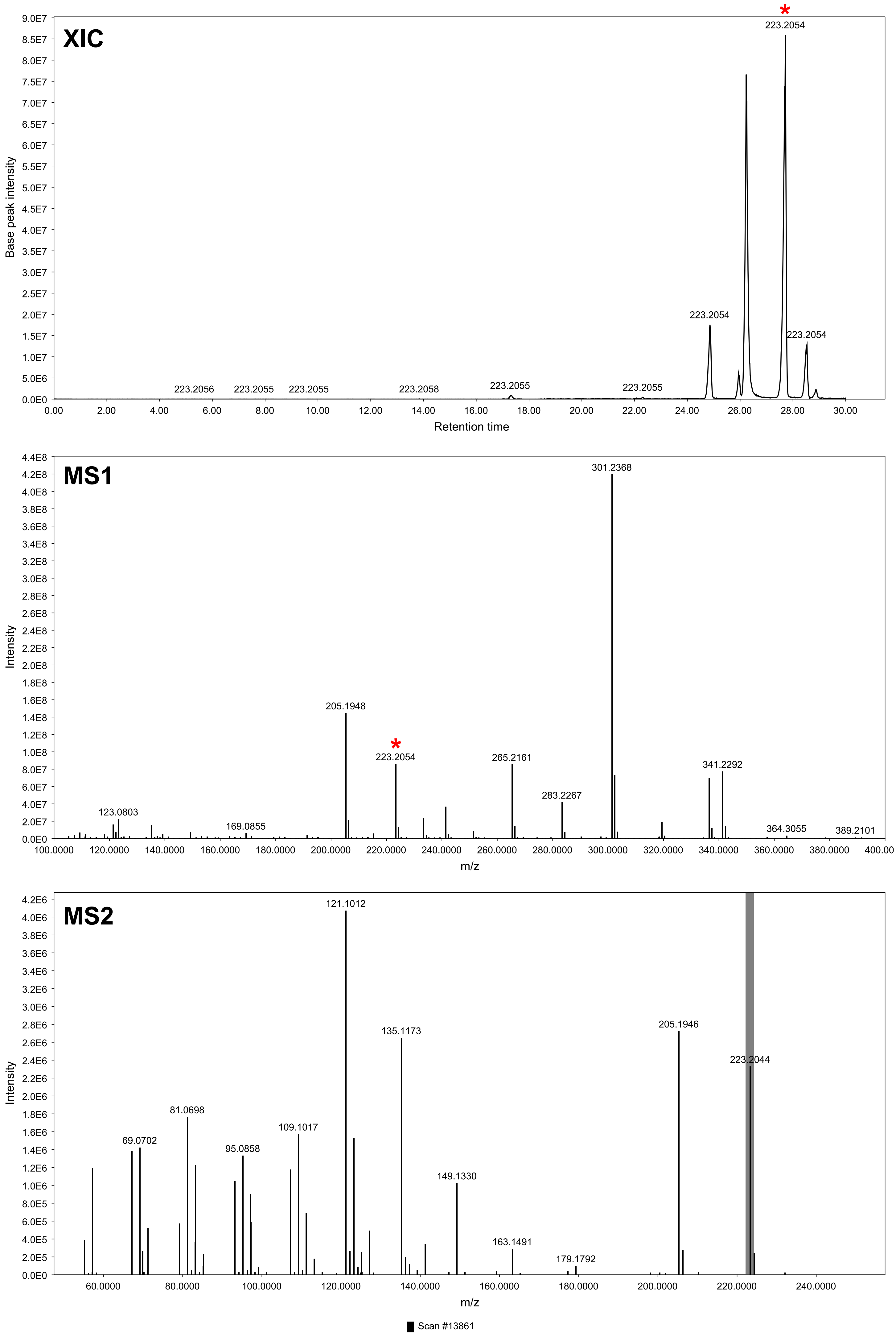

GNPS hit 4 (MEB) - XIC range m/z 205.194-205.196 [M-H2O+H] [M+H]

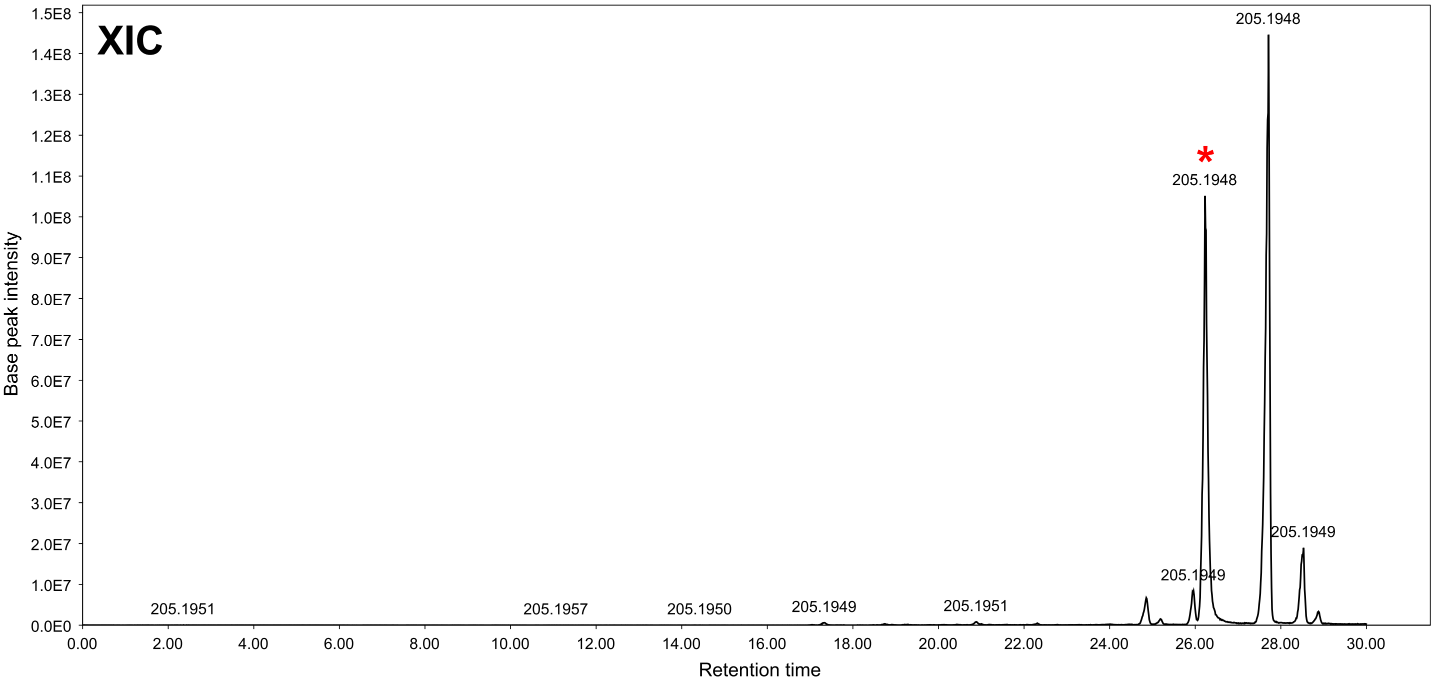

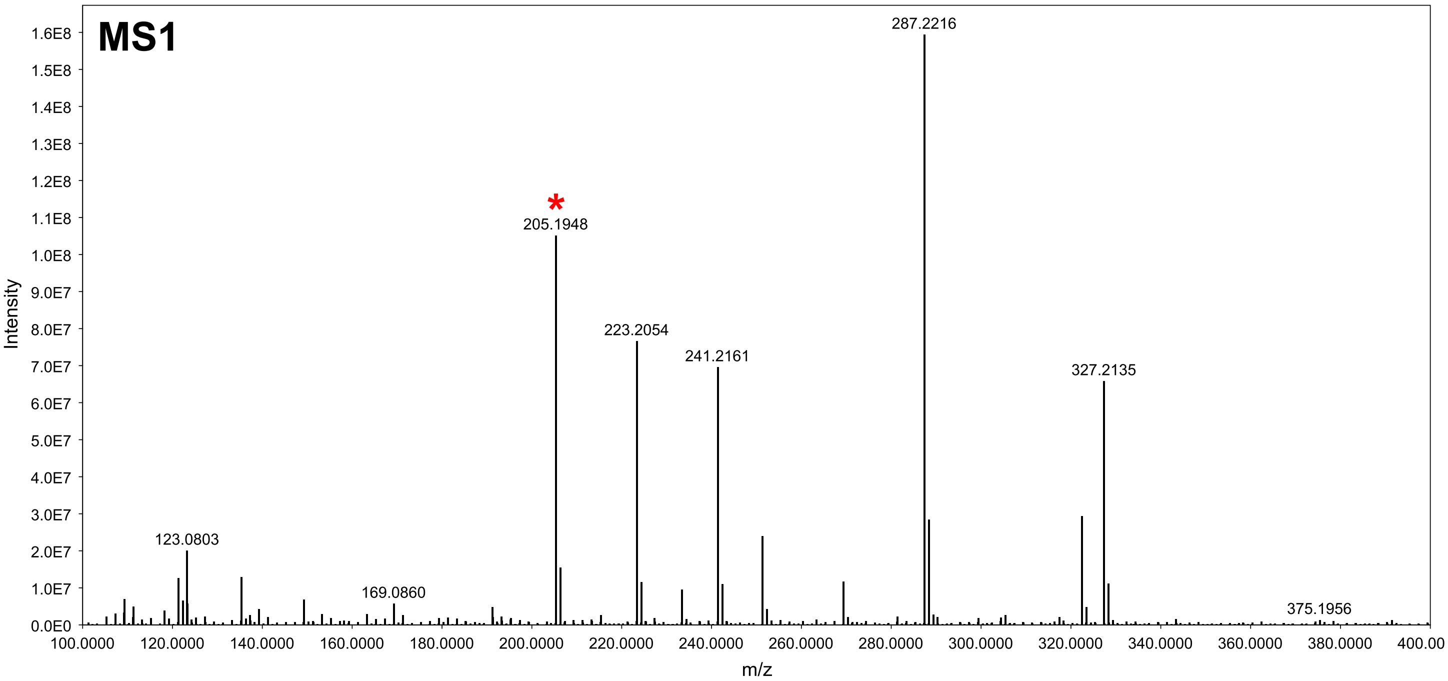

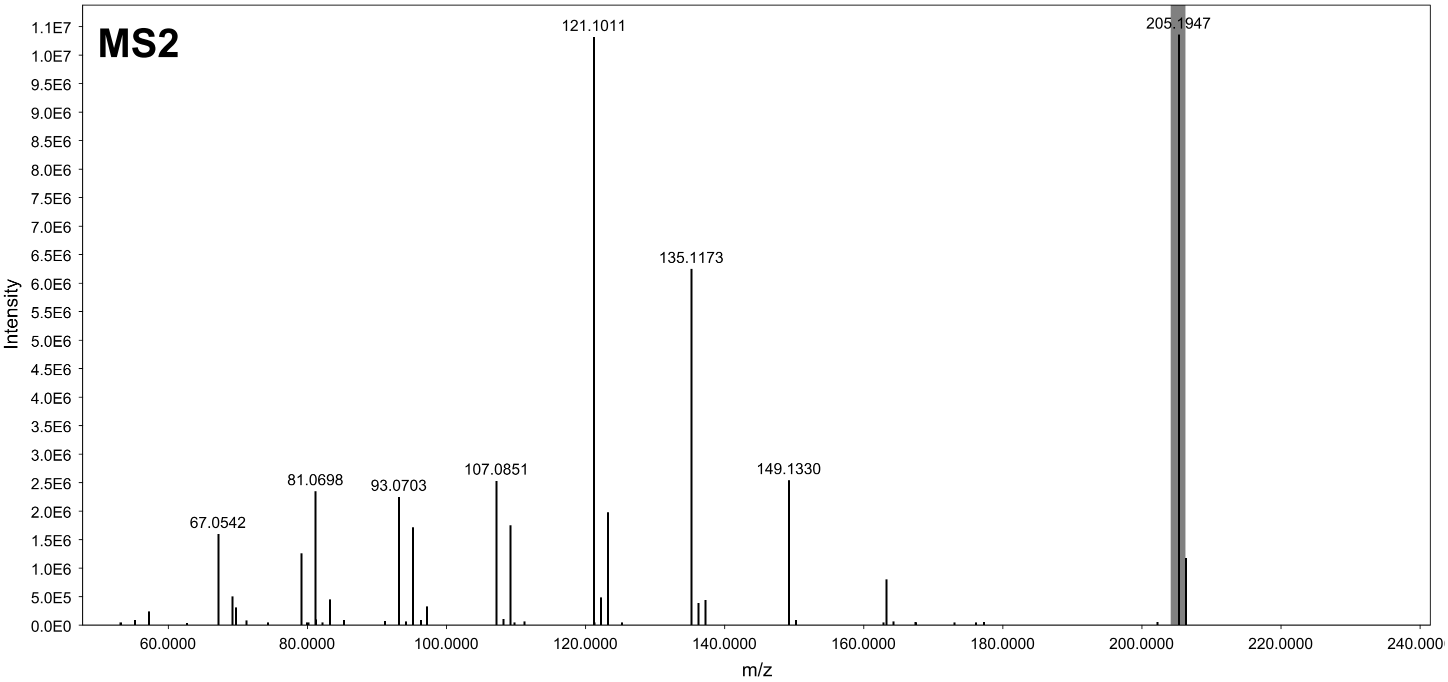

GNPS hit 5a (MEB) - XIC range m/z 203.179-203.181 [M-H2O+H]

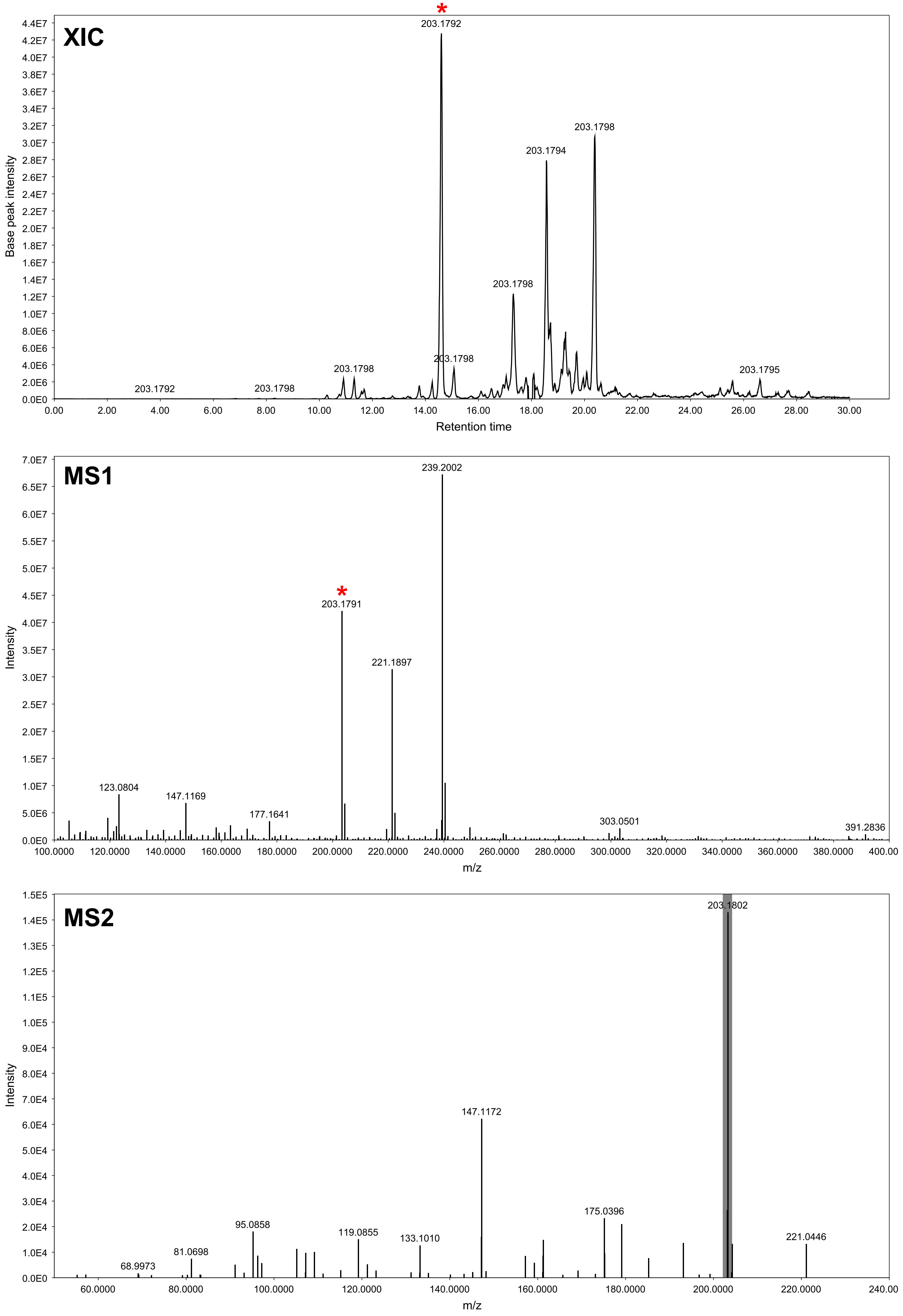

GNPS hit 5b (MEB) - XIC range m/z 203.179-203.181 [M-H2O+H]

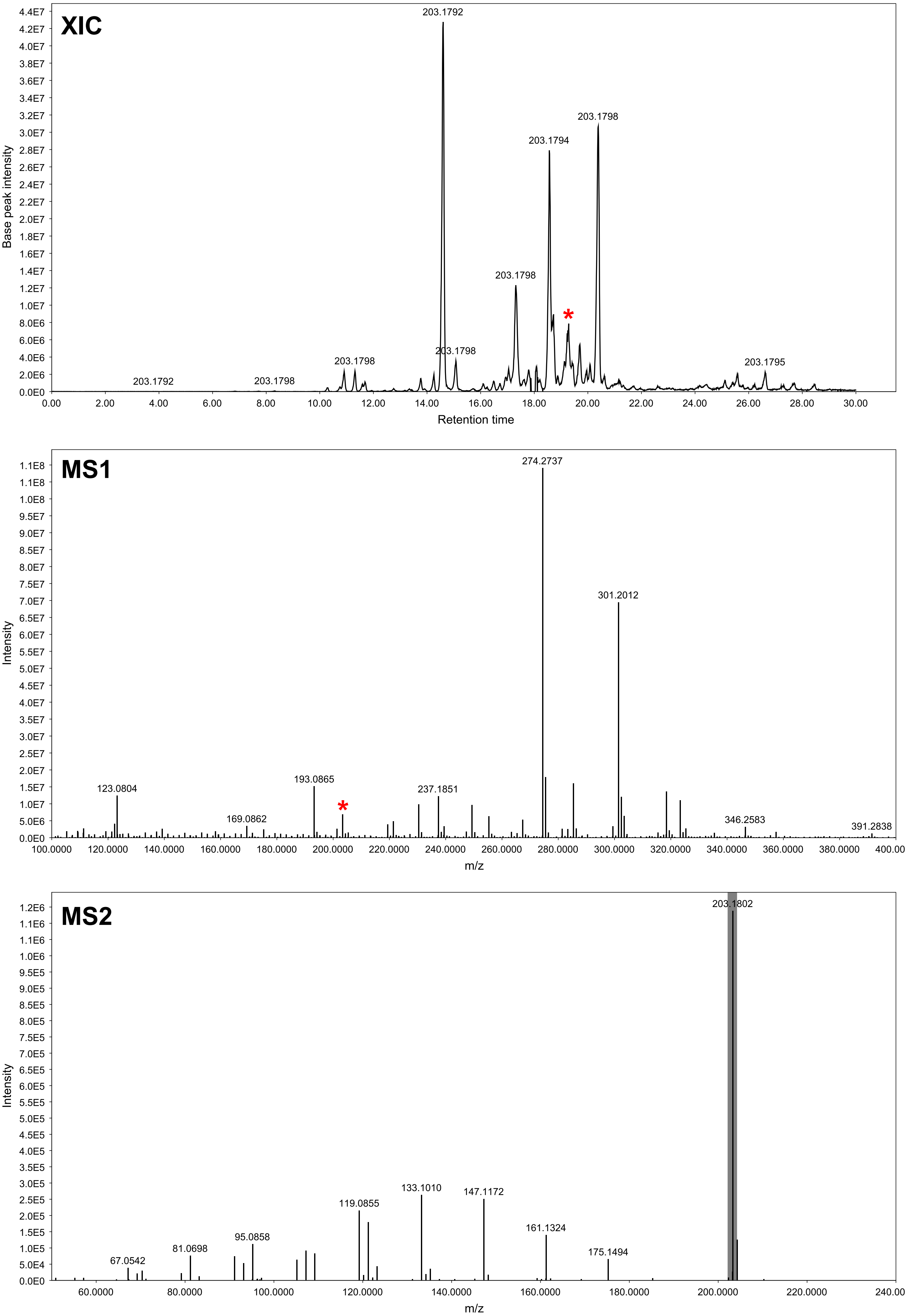

GNPS hit 5c (MEB) - XIC range m/z 203.179-203.181 [M-H2O+H]

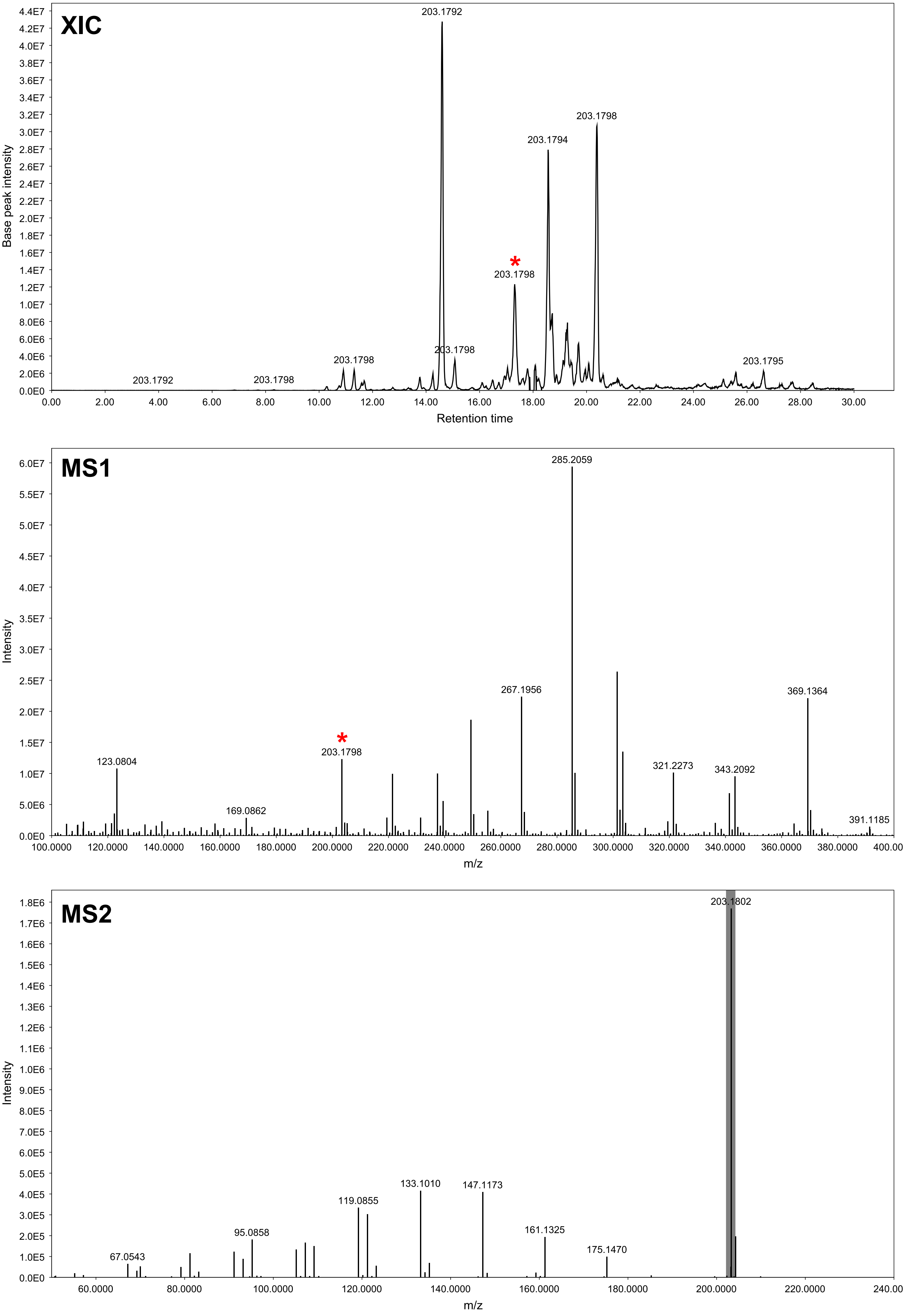

GNPS hit 6a (PDA) - XIC range m/z 201.163-201.165 [$M-2H_2O+H$]

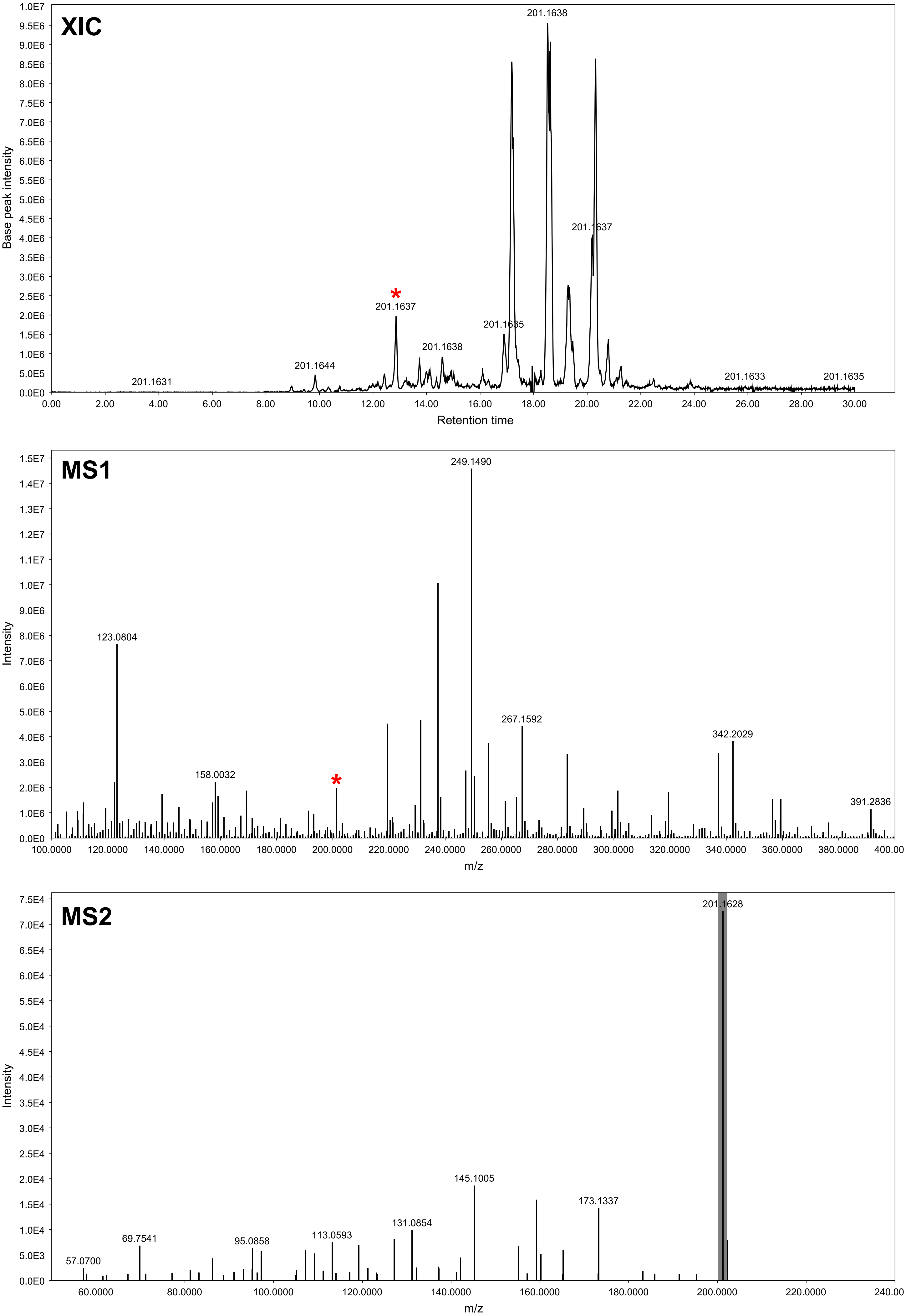

GNPS hit 6b (PDA) - XIC range m/z 219.172-219.174 [M-H_2O+H]

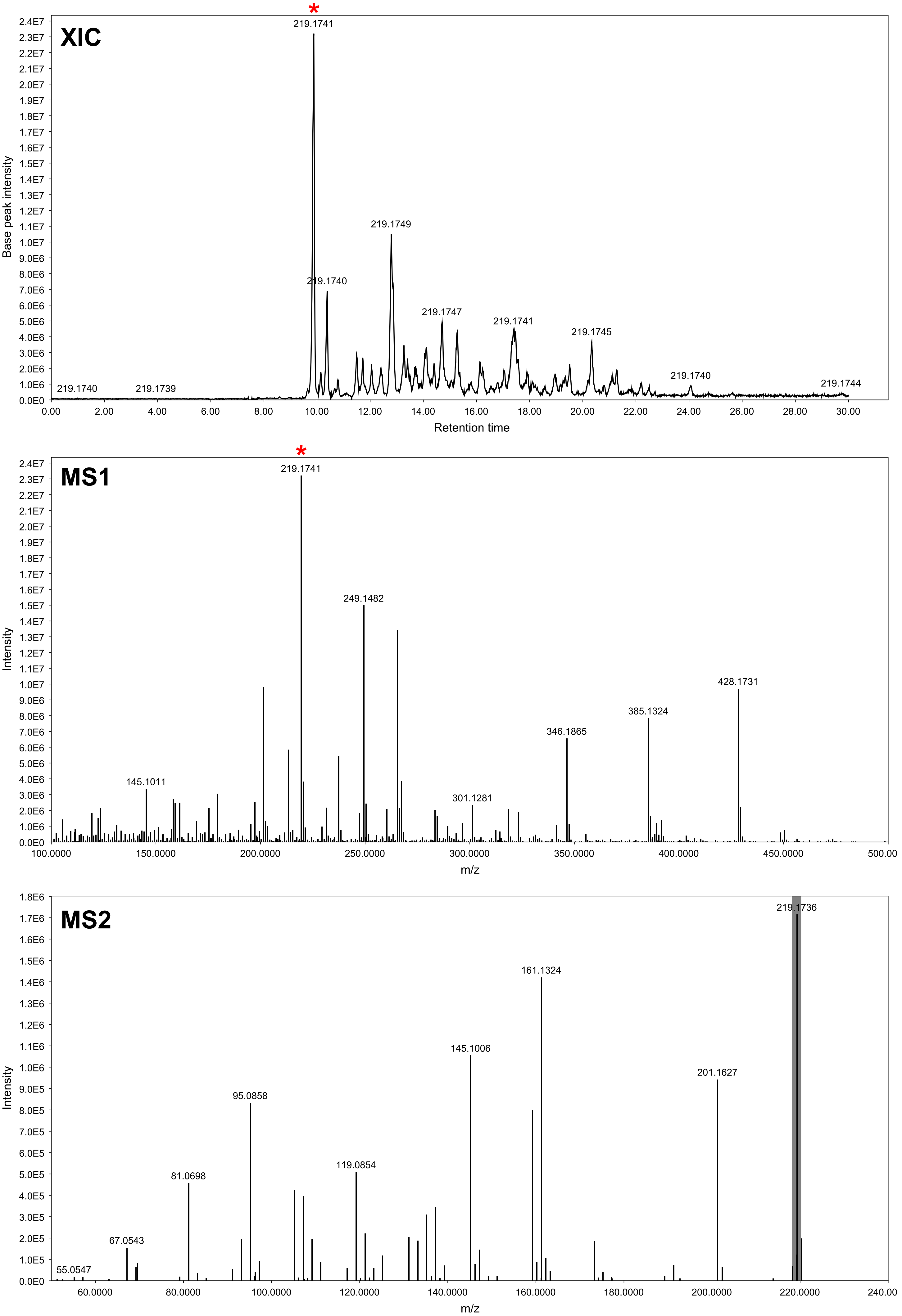

GNPS hit 6c (MEB) - XIC range m/z 237.184-237.186 [M+H]

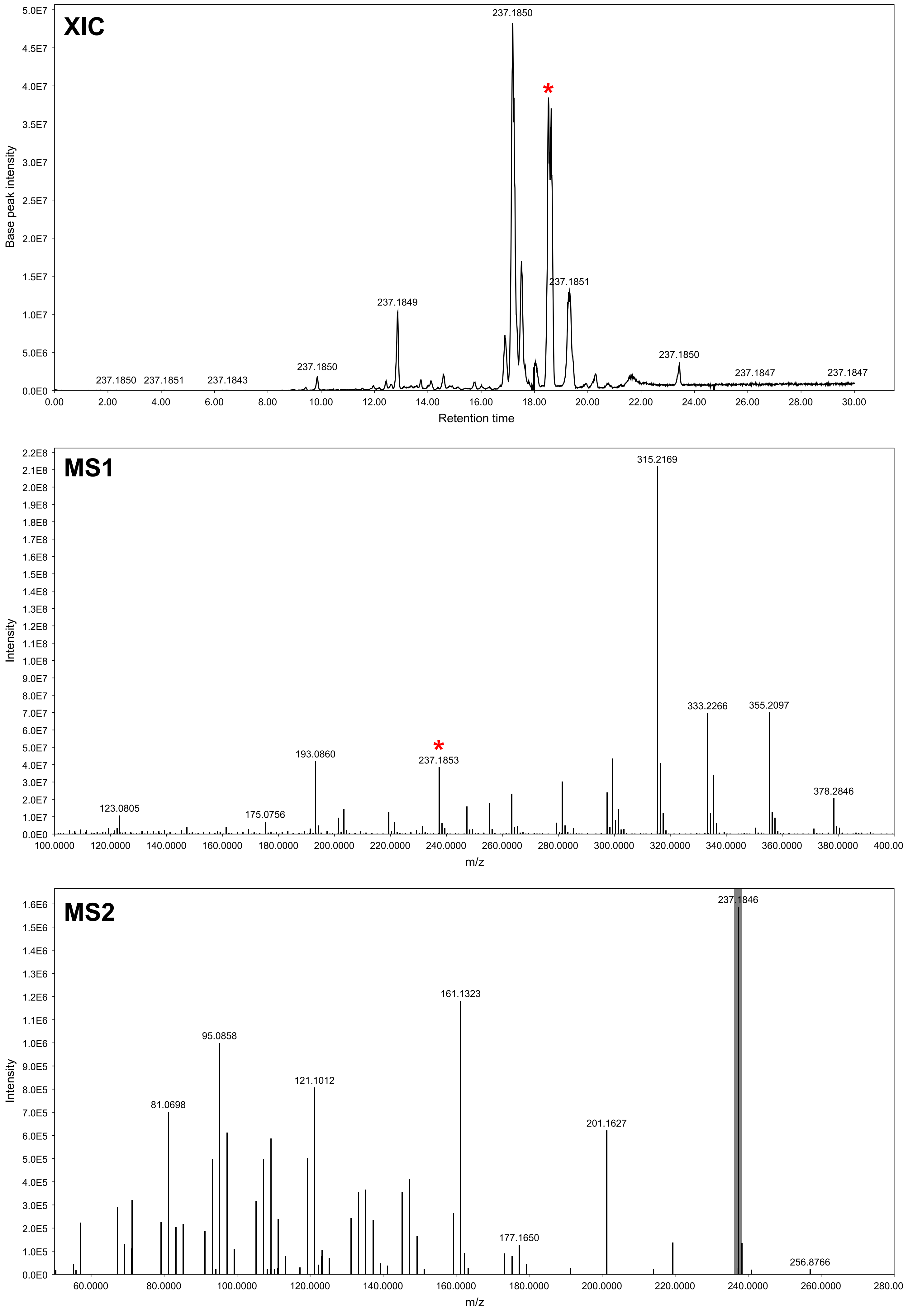

GNPS hit 7 (MEB) - XIC range m/z 235.168-235.170 [M+H]

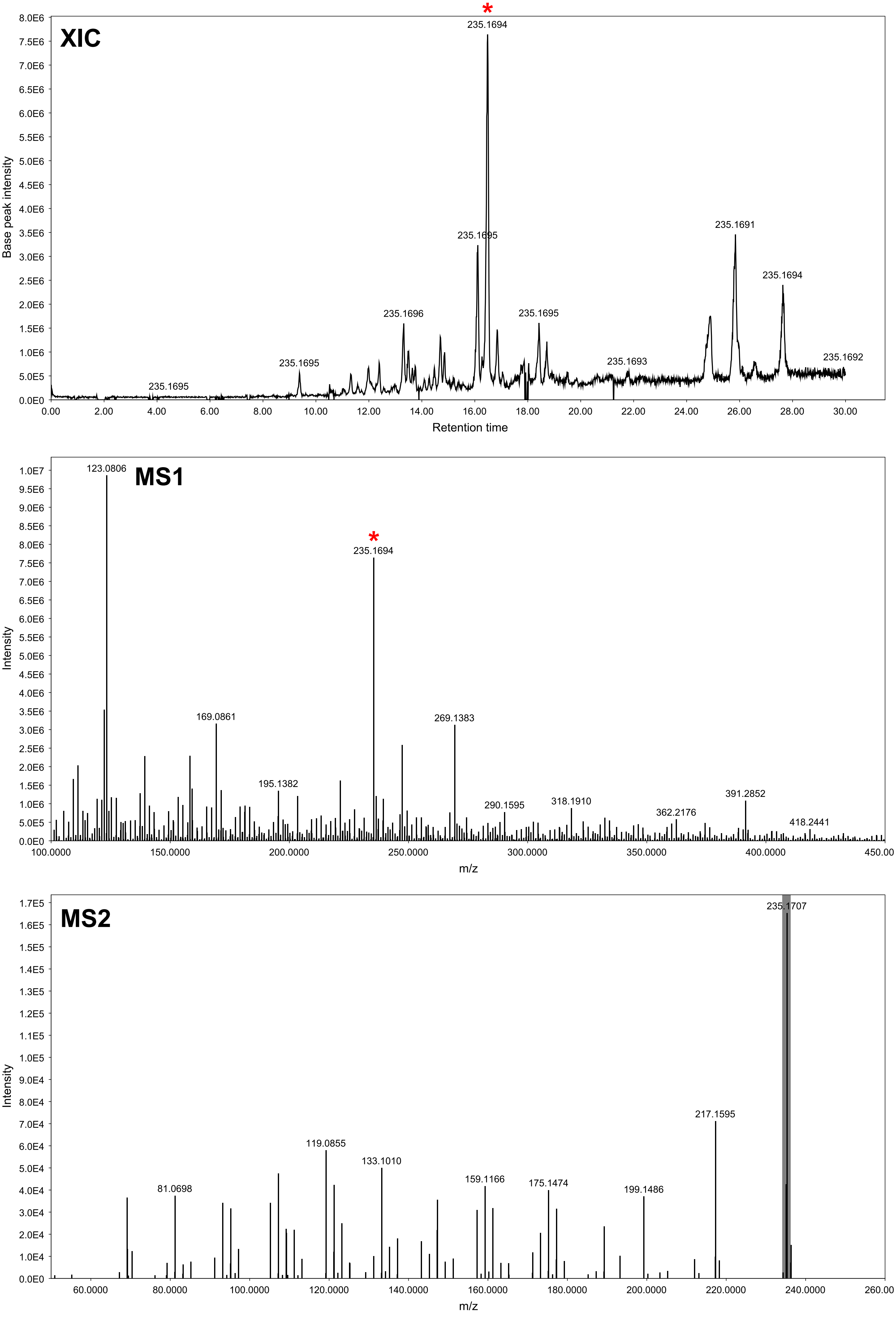

GNPS hit 8 (MEB) - XIC range m/z 235.168-235.170

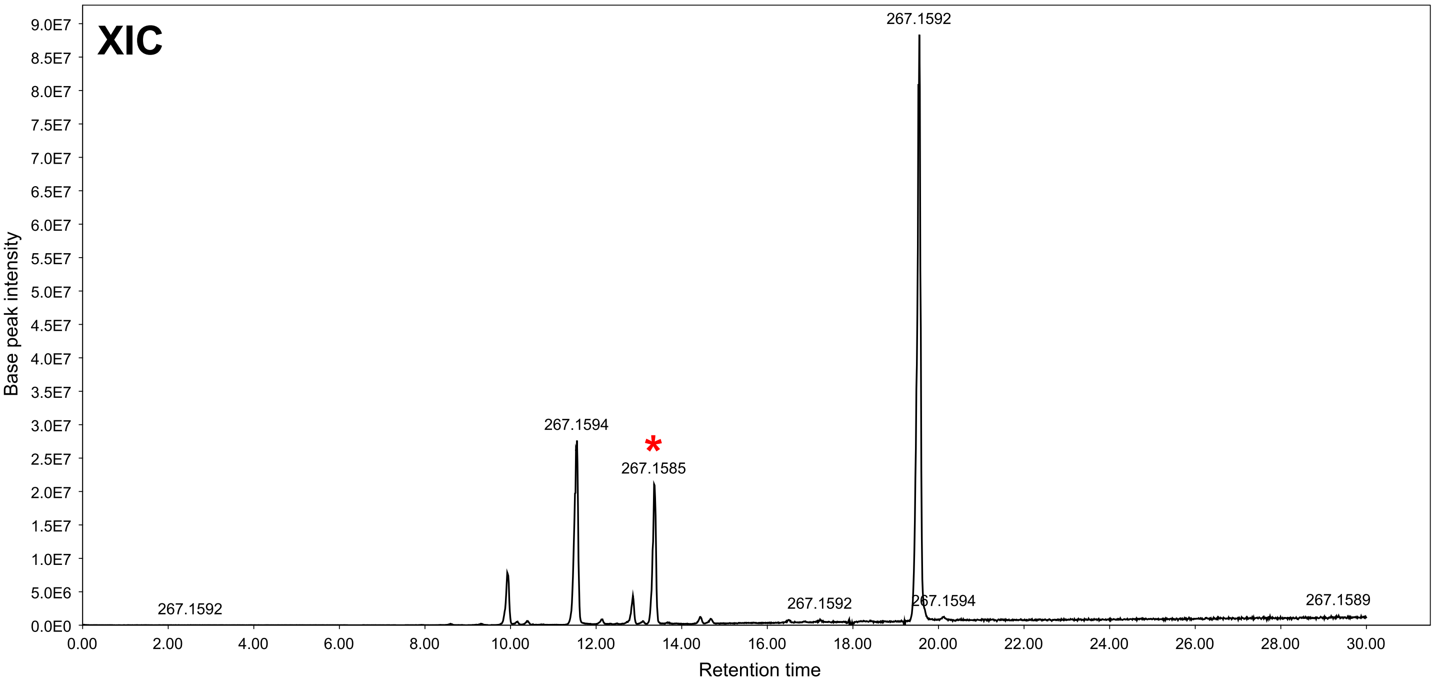

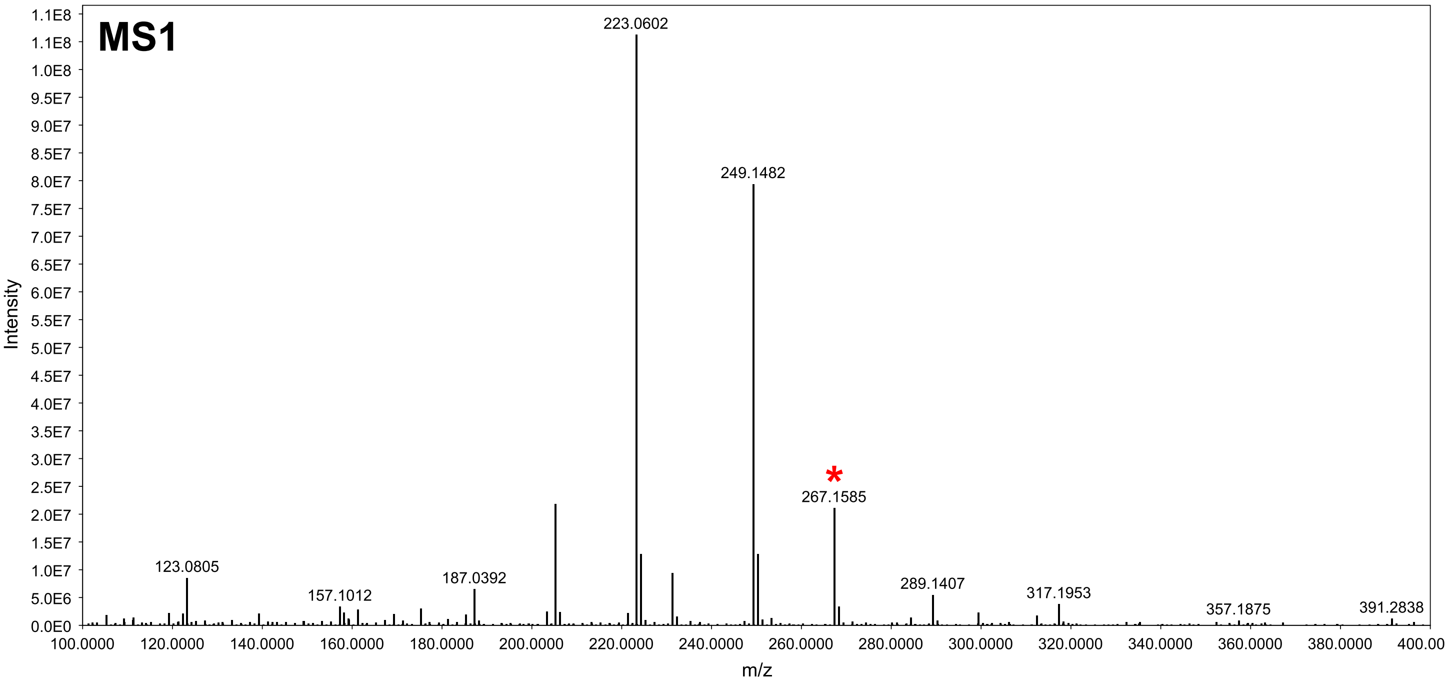

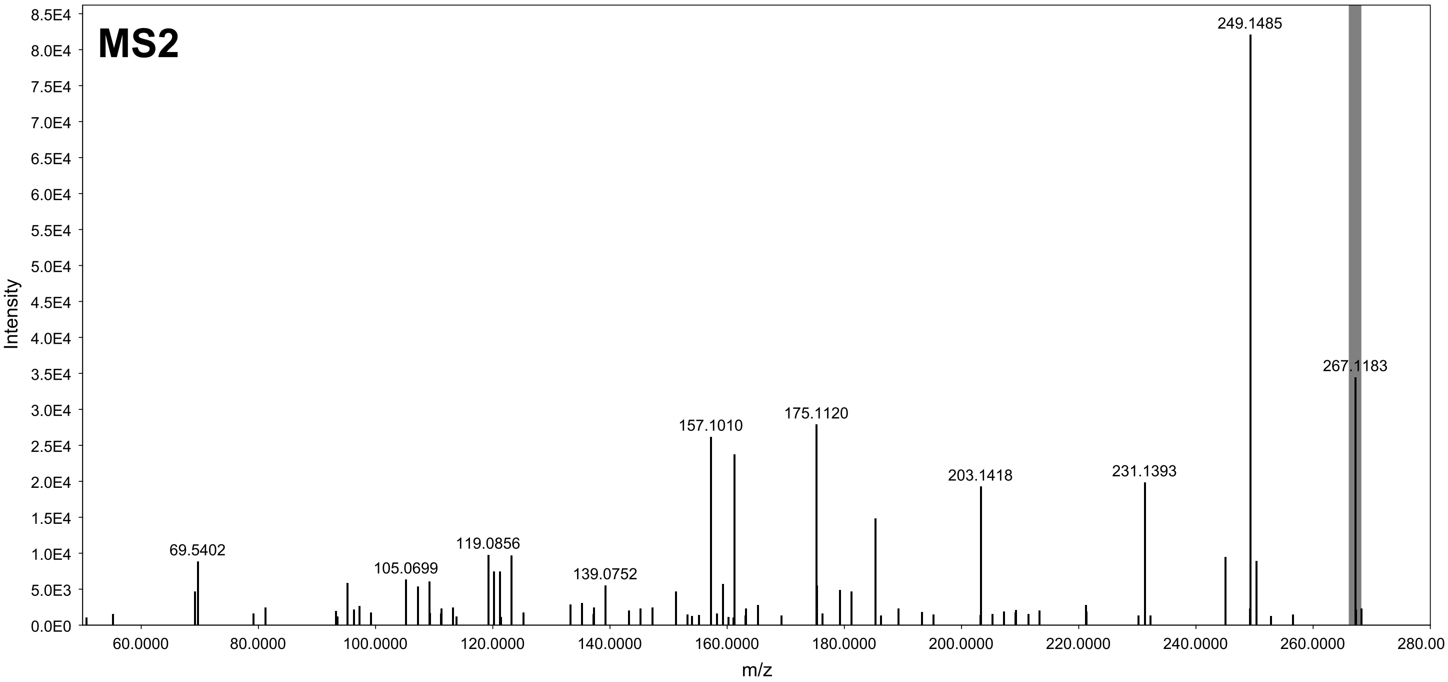

GNPS hit 9a (PDA) - XIC range m/z 265.142-265.144 [M+H]

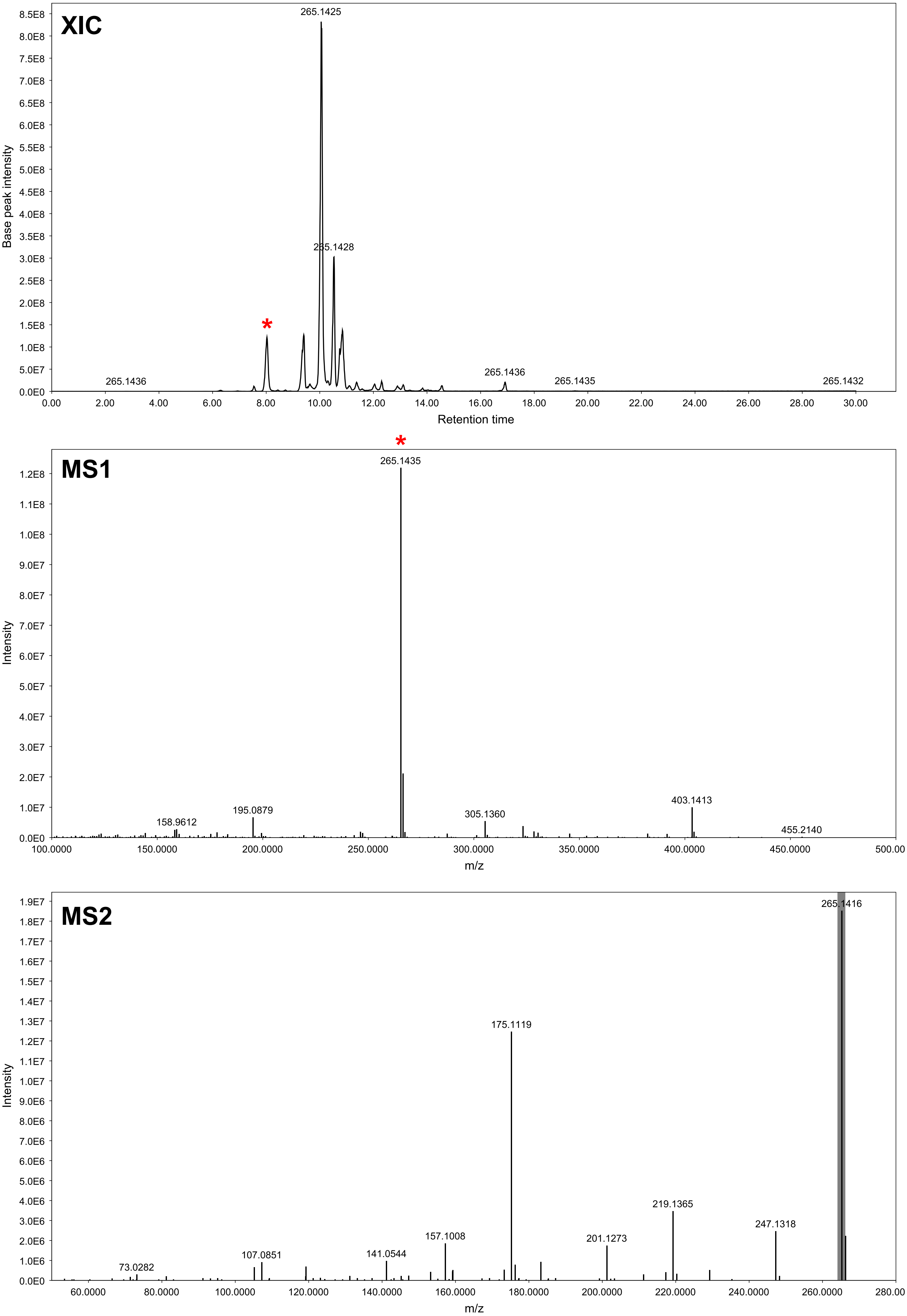

GNPS hit 9b (PDA) - XIC range m/z 265.142-265.144 [M+H]

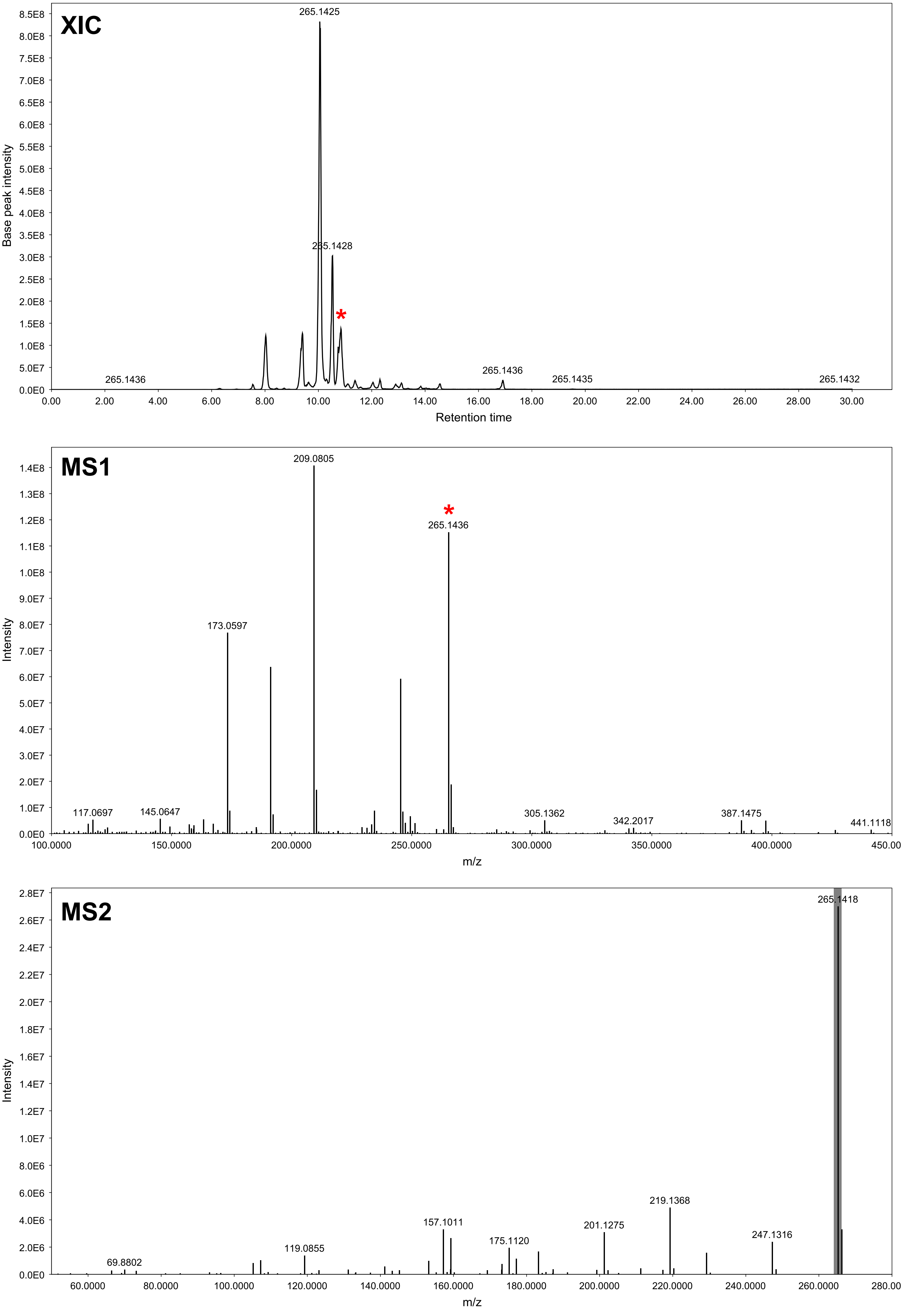

GNPS hit 9c (MEB) - XIC range m/z 265.142-265.144 [M+H]

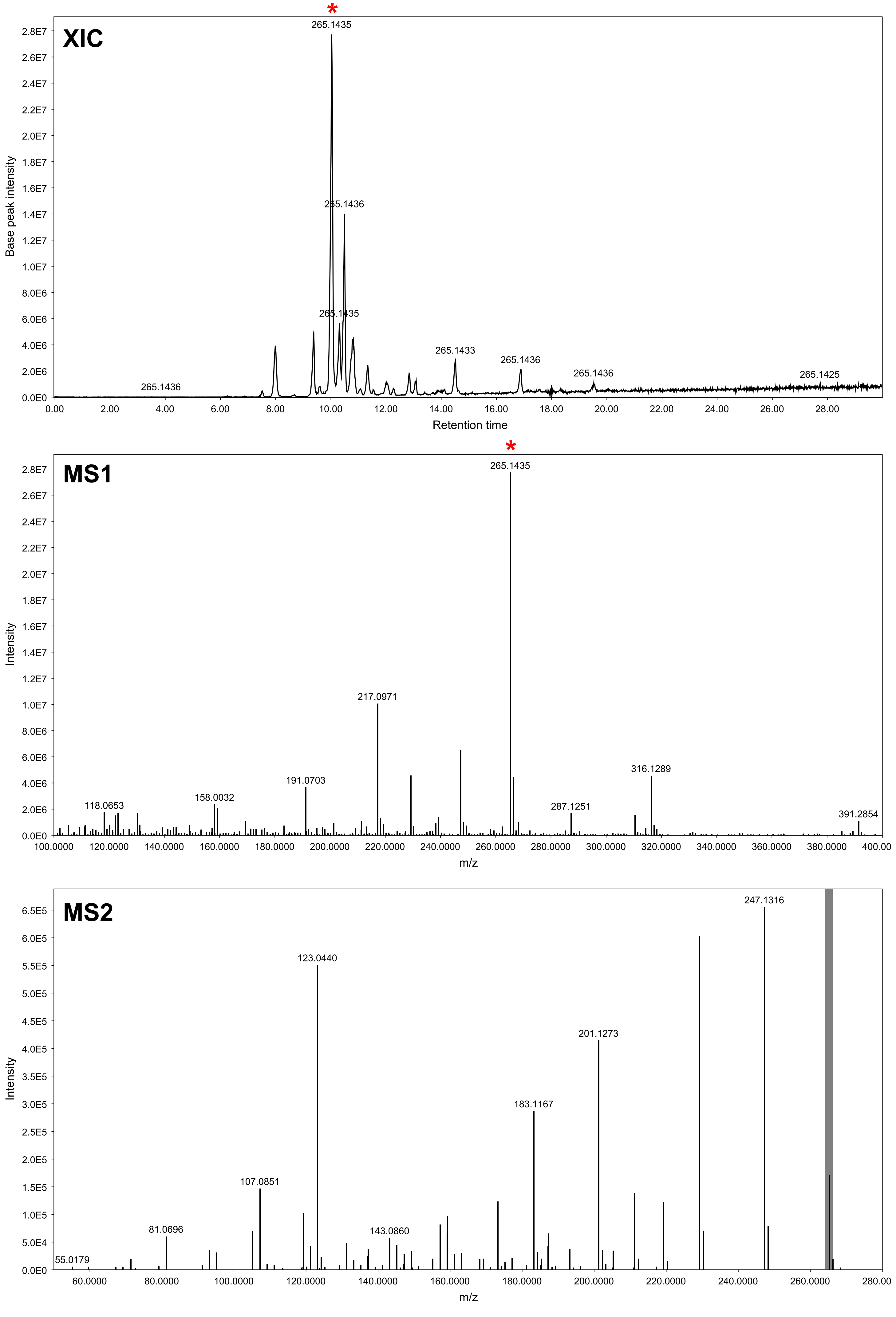

GNPS hit 9d (MEB) - XIC range m/z 265.142-265.144 [M+H]

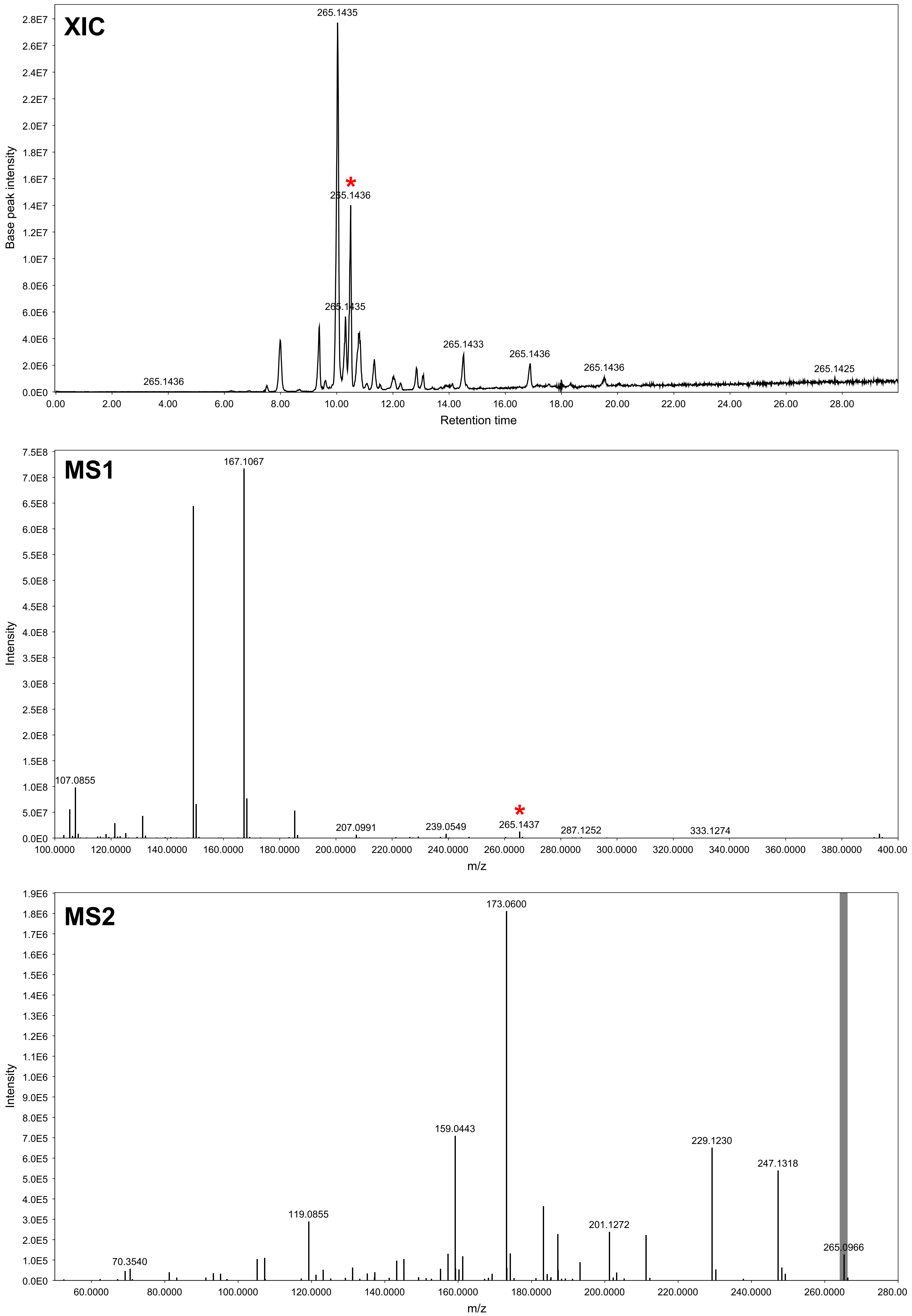

GNPS hit 10 (PDA) - XIC range m/z 265.142-265.144 [M+H]

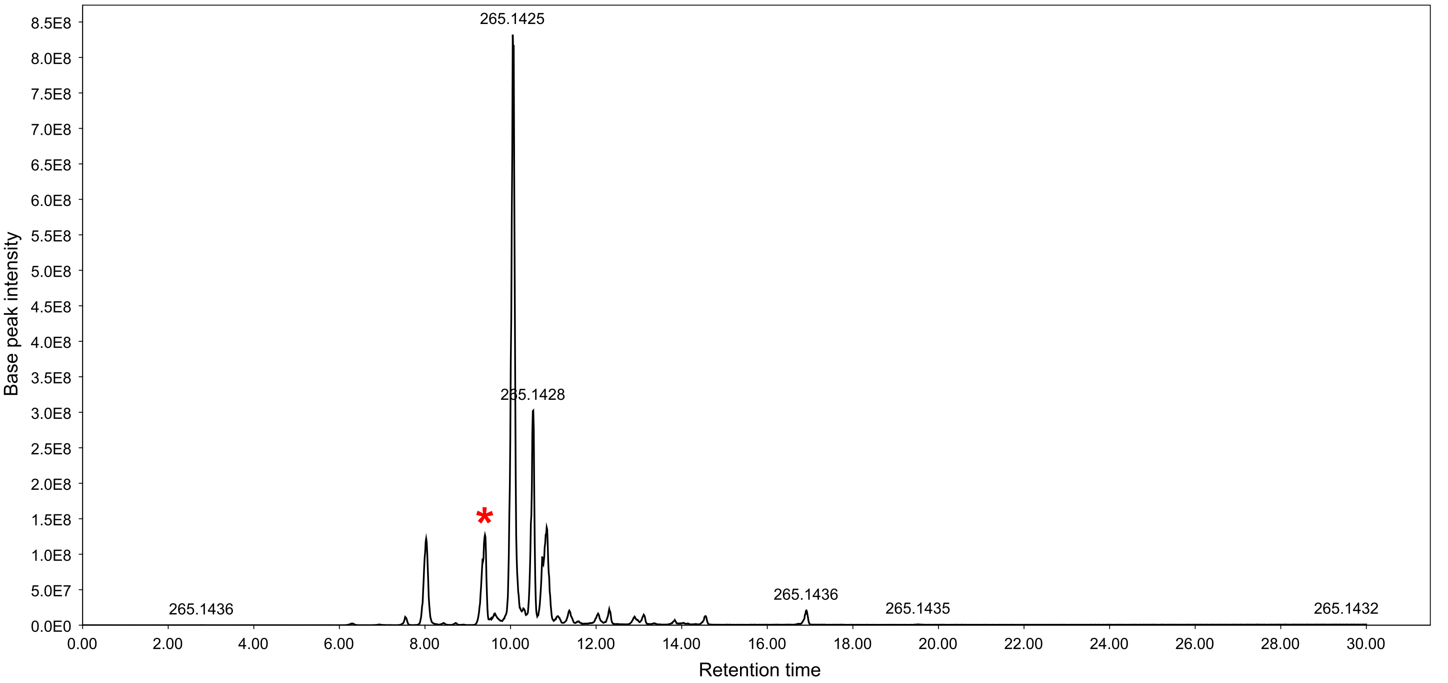

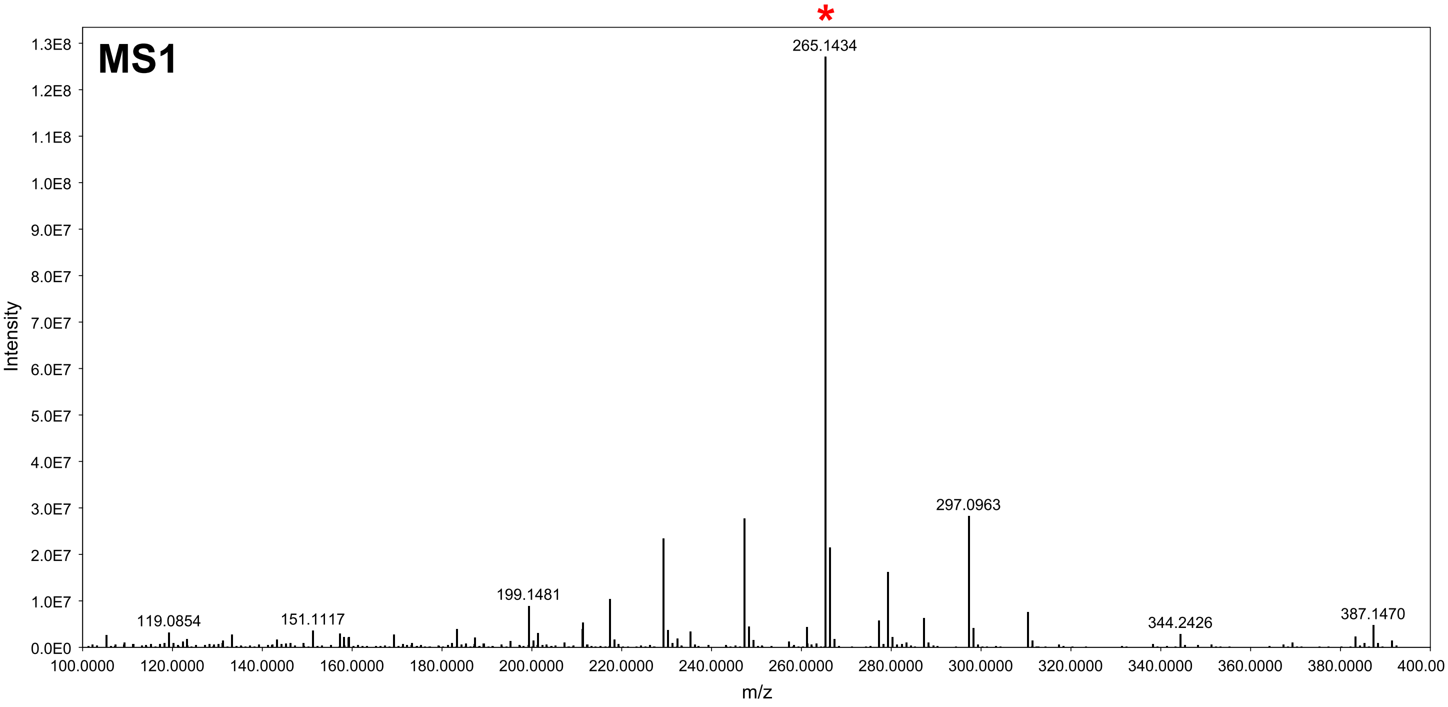

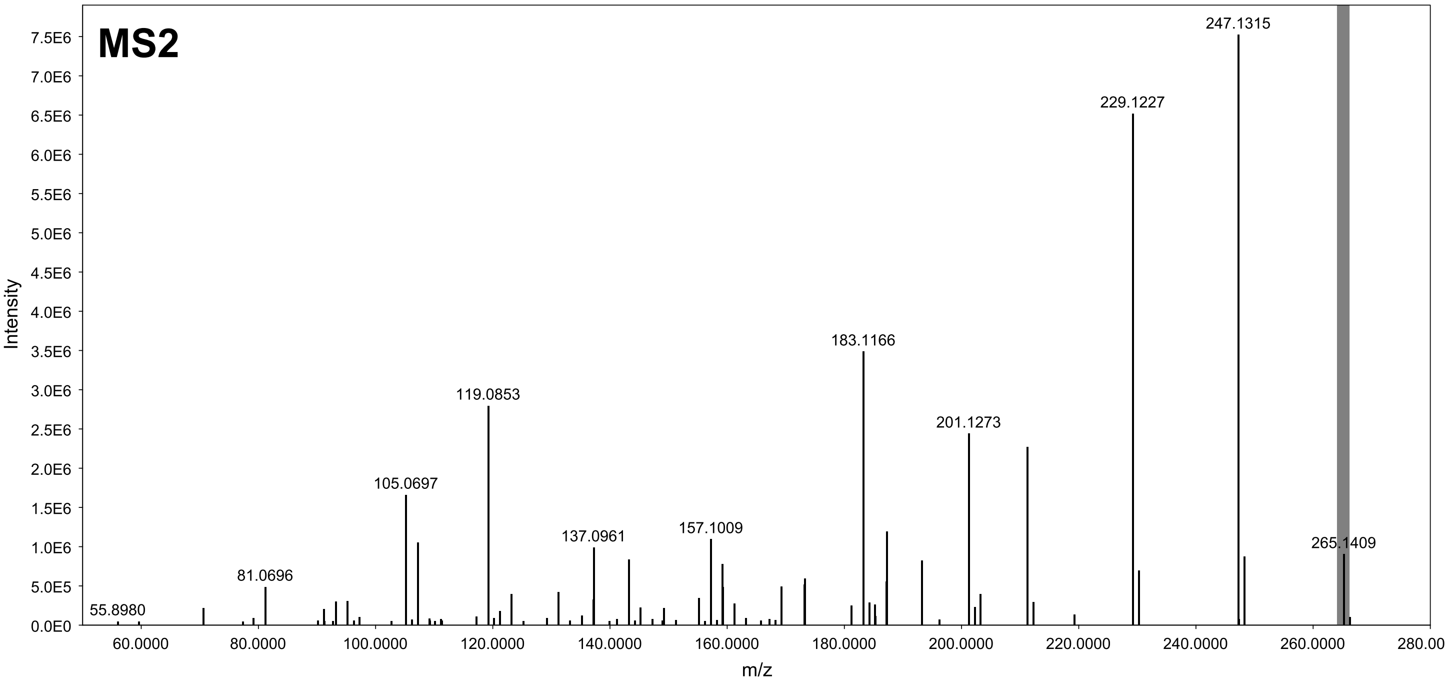

GNPS hit 11a (MEB) - XIC range m/z 231.137-231.139 [M-H2O+H]

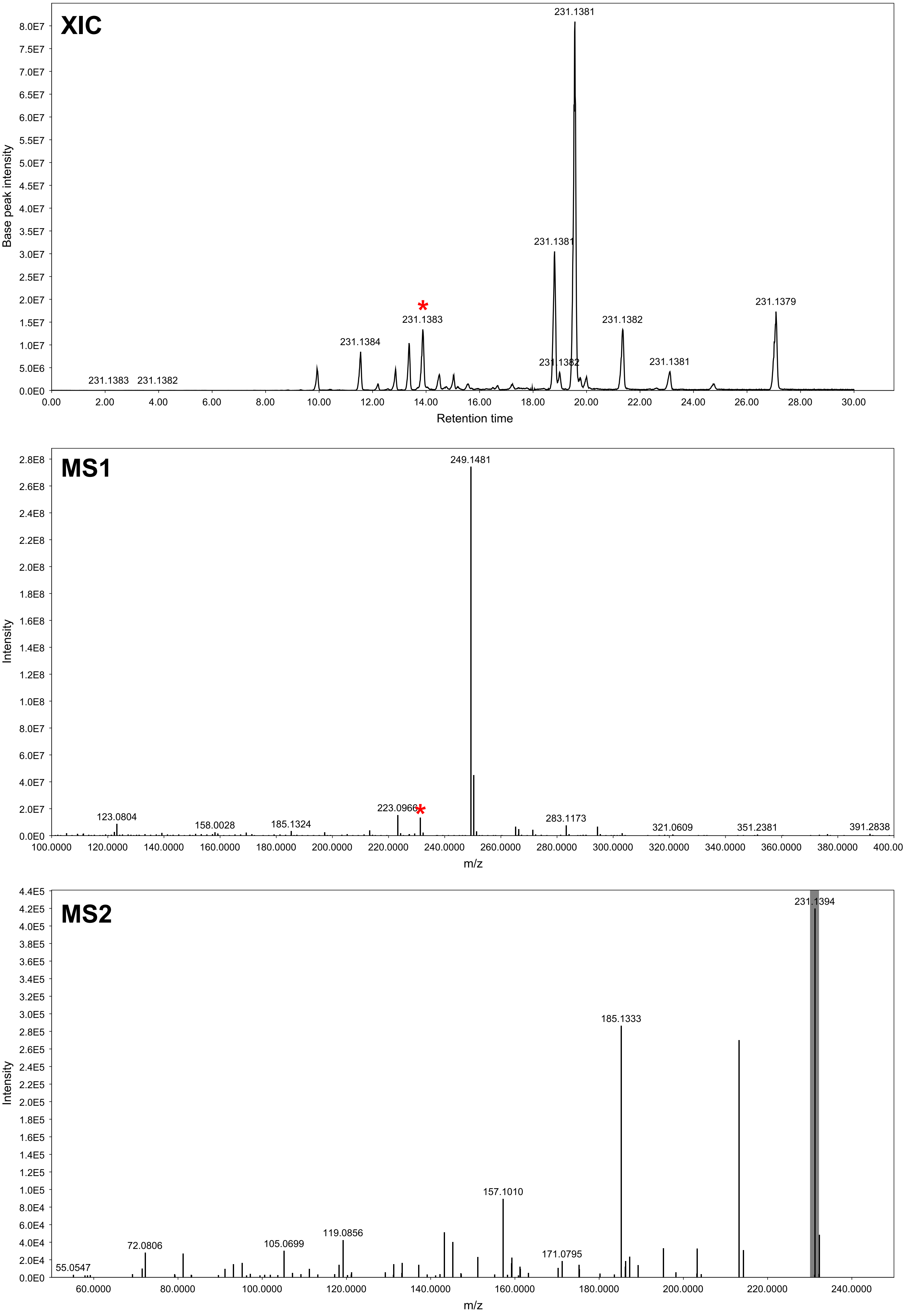

GNPS hit 11b (MEB) - XIC range m/z 231.137-231.139 [M-H2O+H]

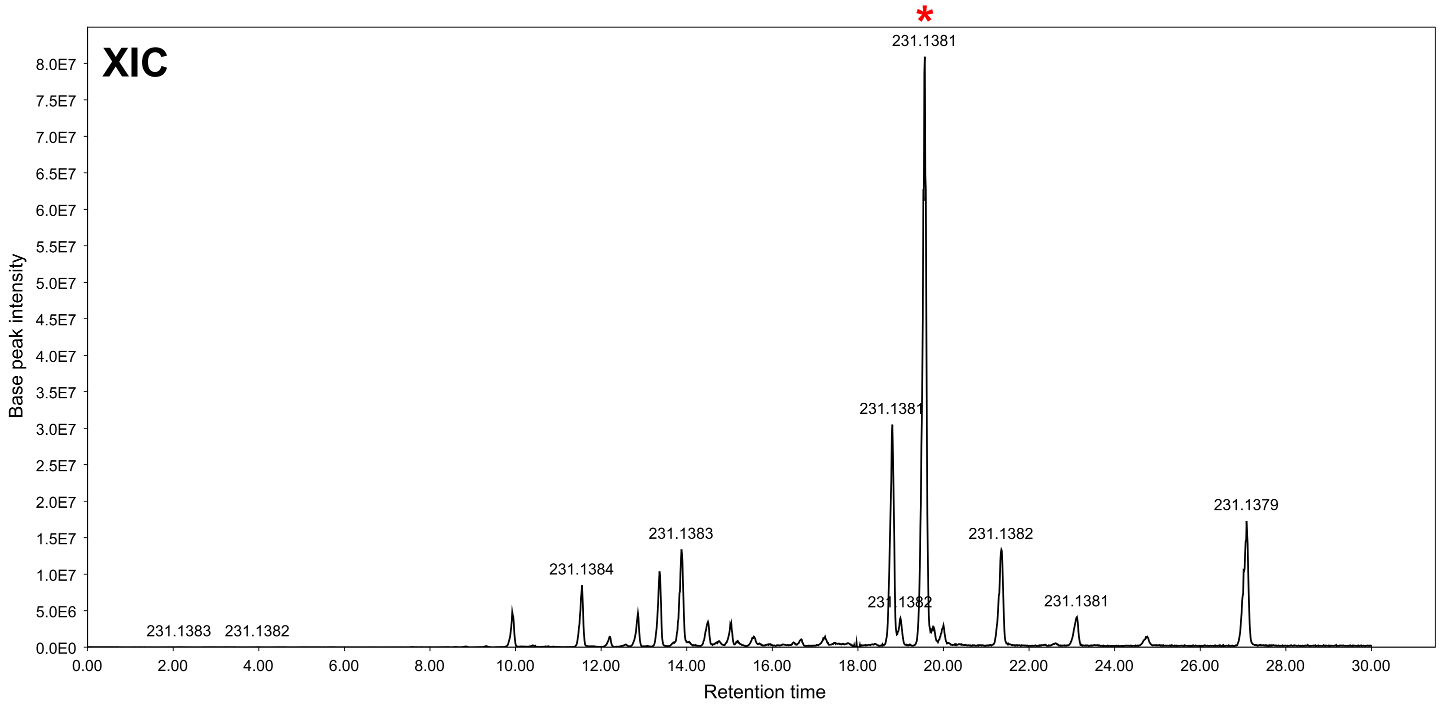

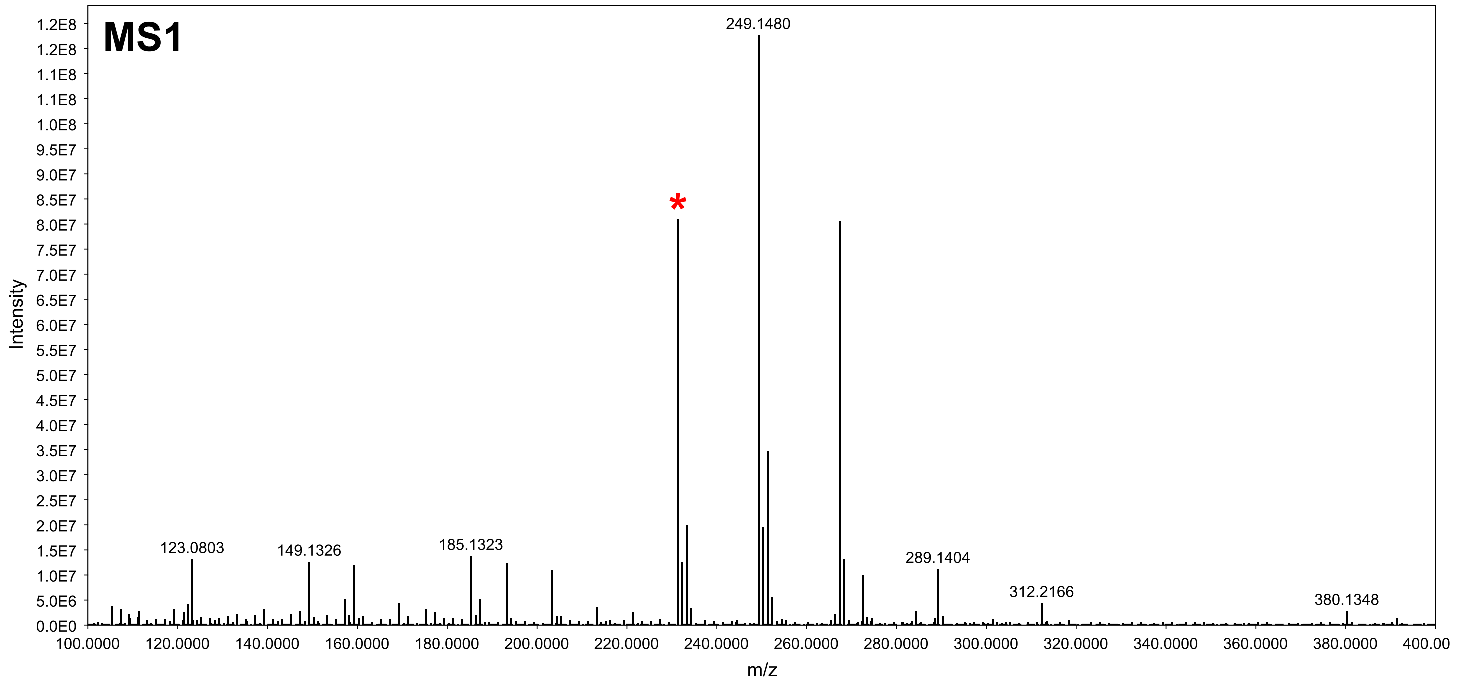

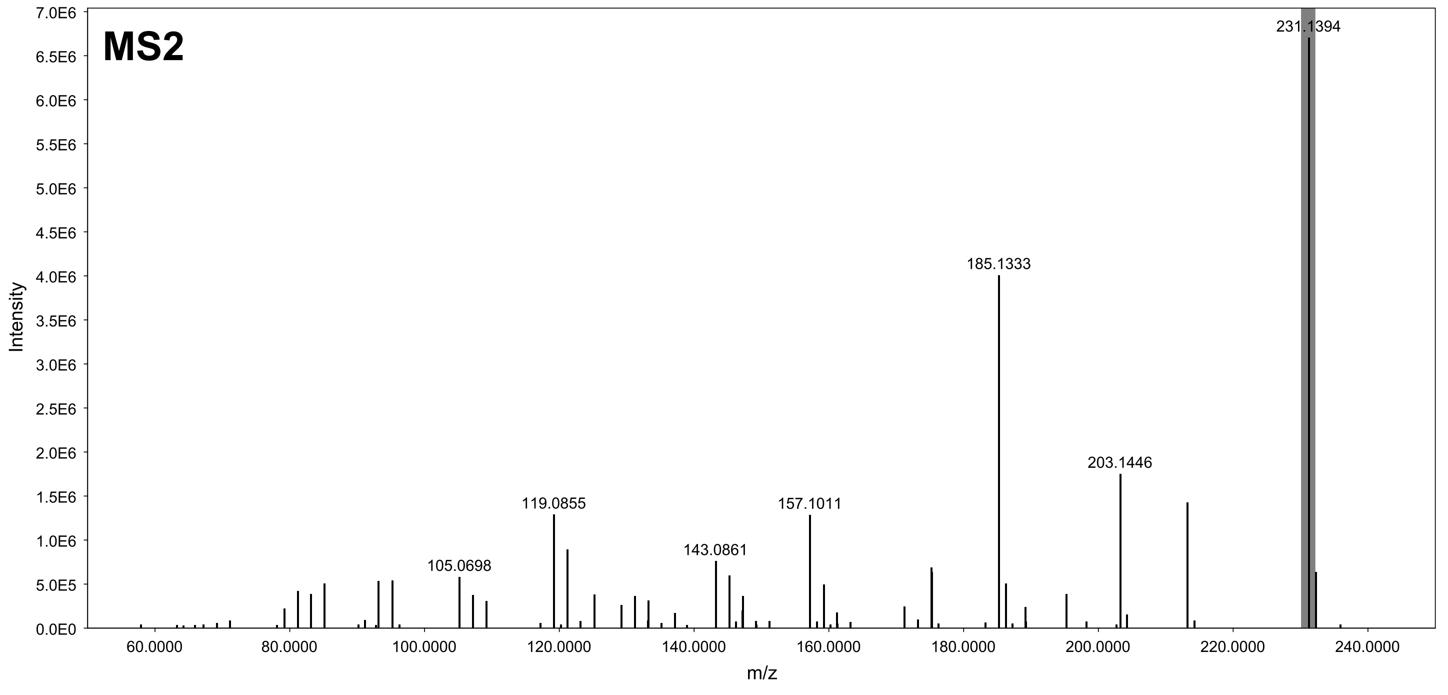

GNPS hit 11c (PDA) - XIC range m/z 231.137-231.139 [M-H2O+H]

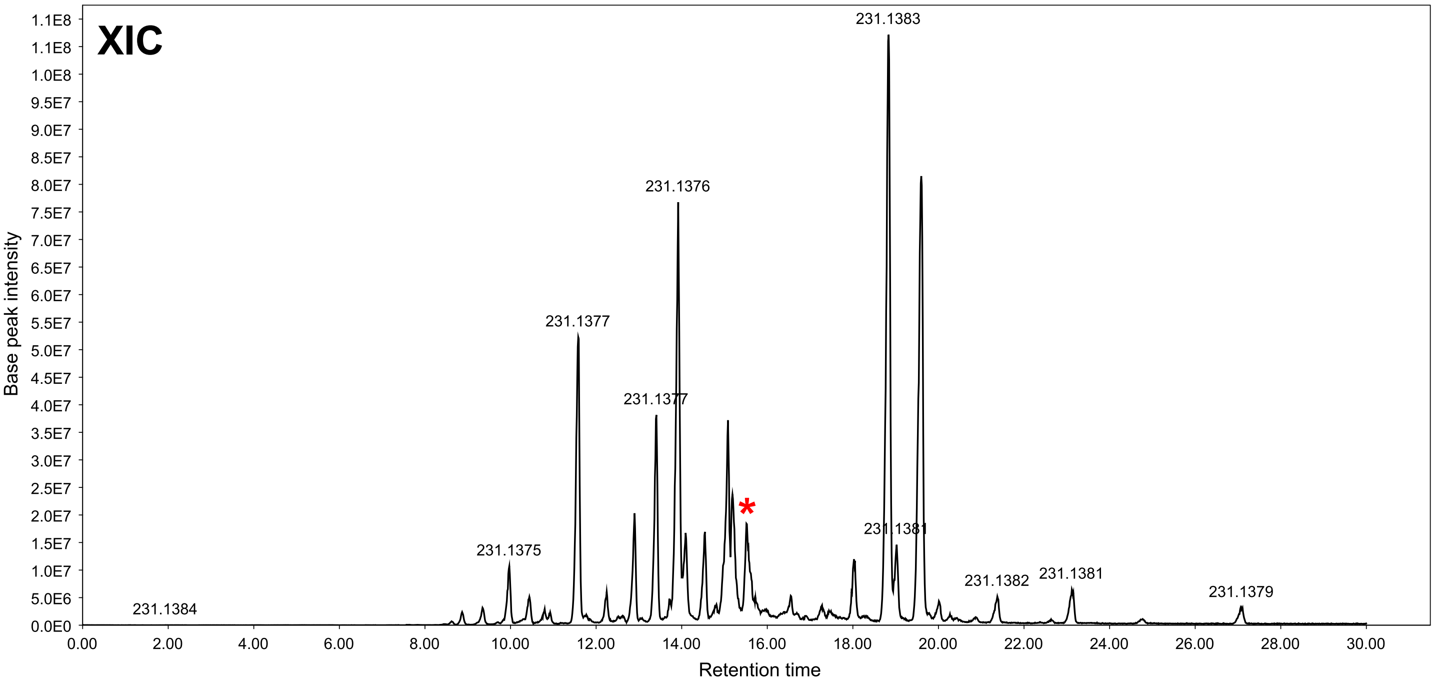

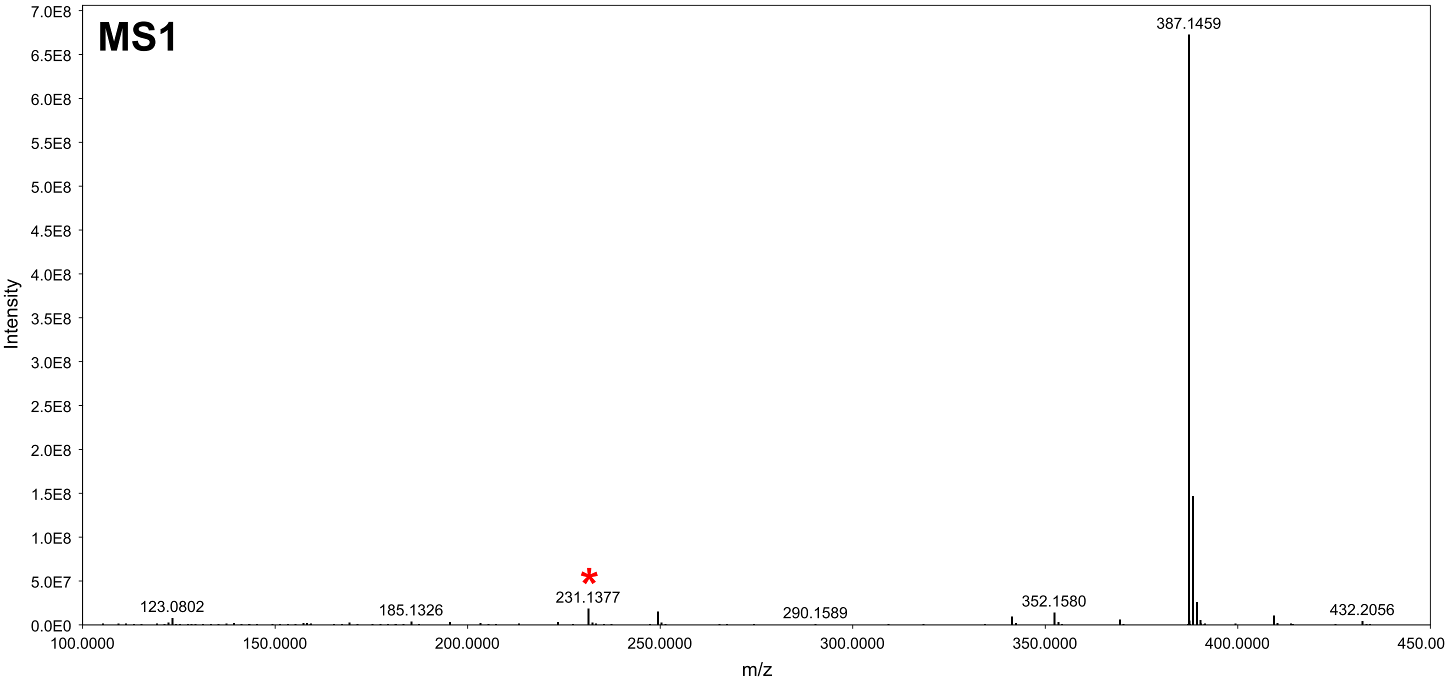

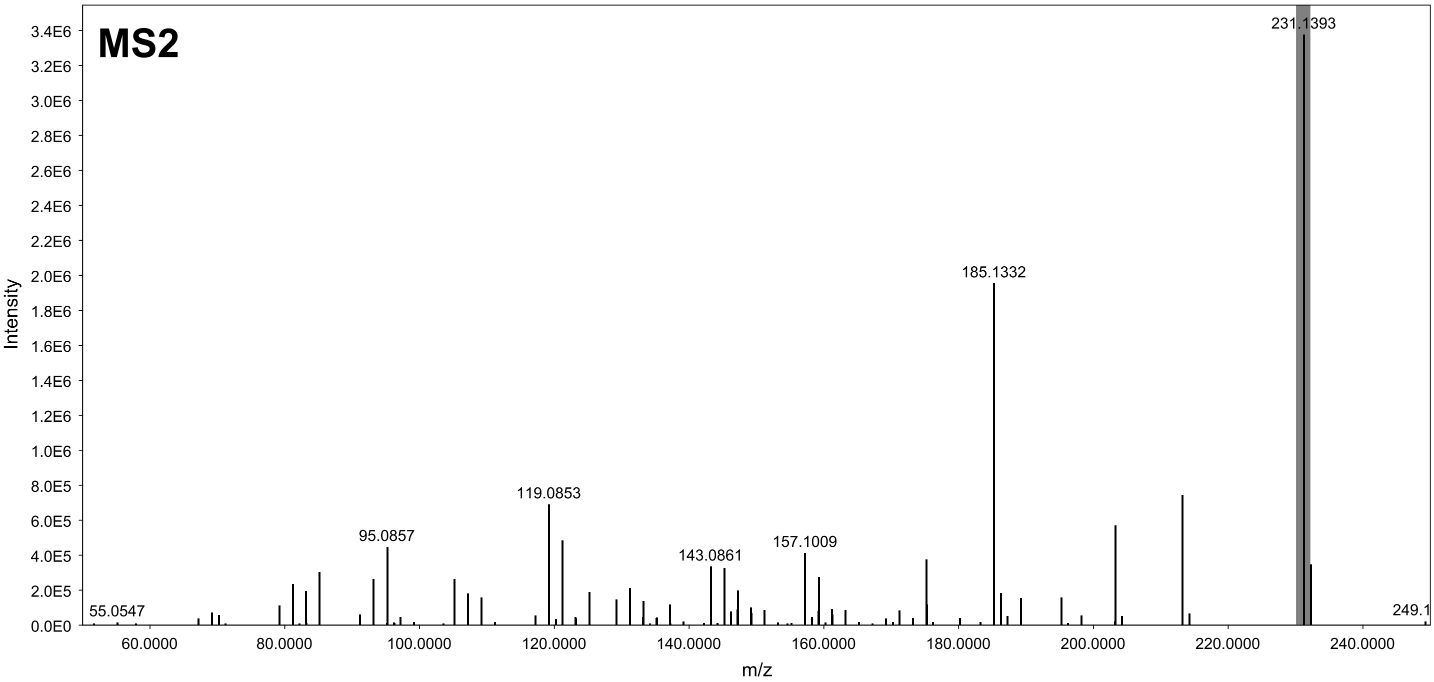

GNPS hit 11d (PDA) - XIC range m/z 231.137-231.139 [M-H2O+H]

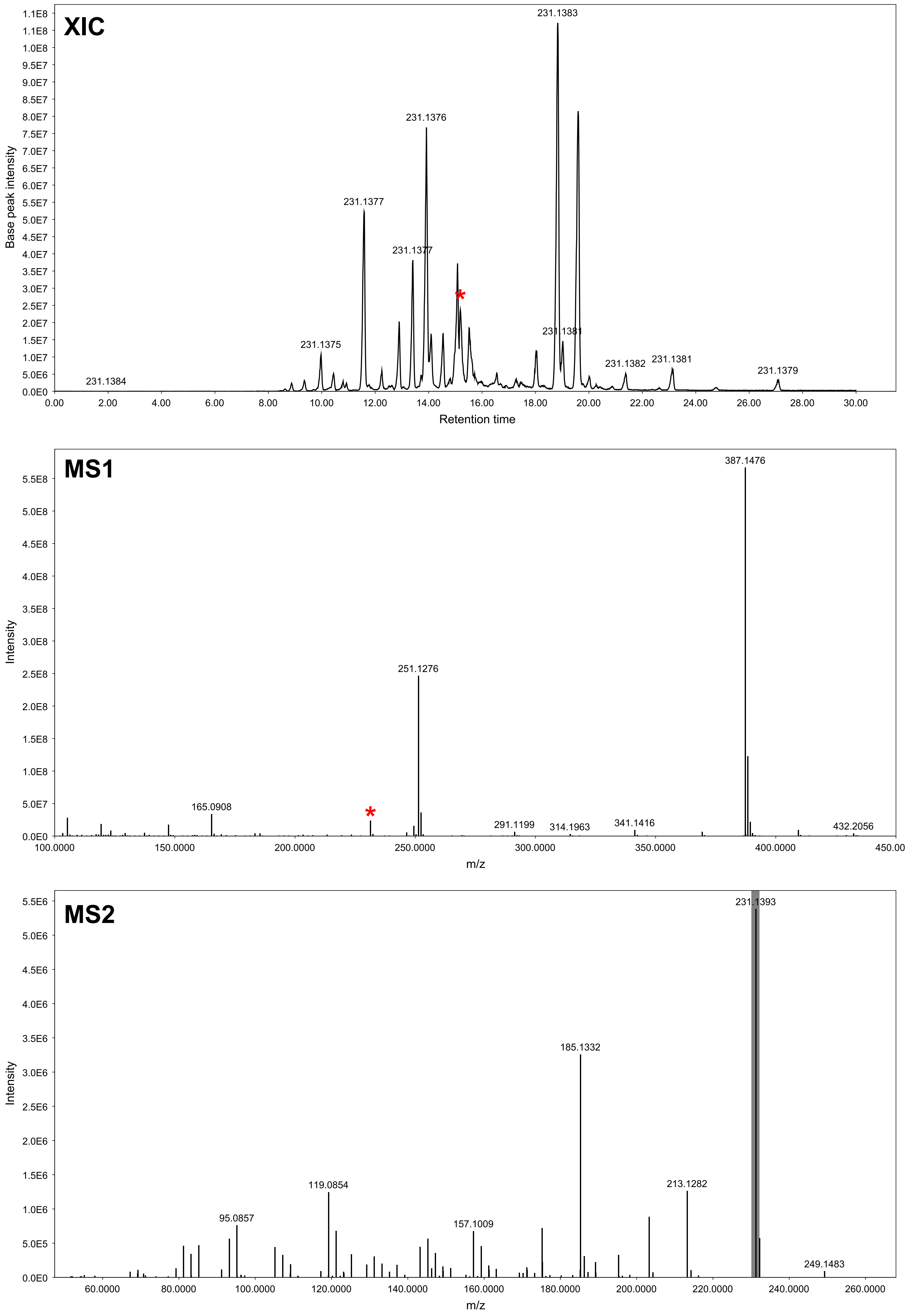

GNPS hit 12a (PDA) - XIC range m/z 231.137-231.139 [M+H]

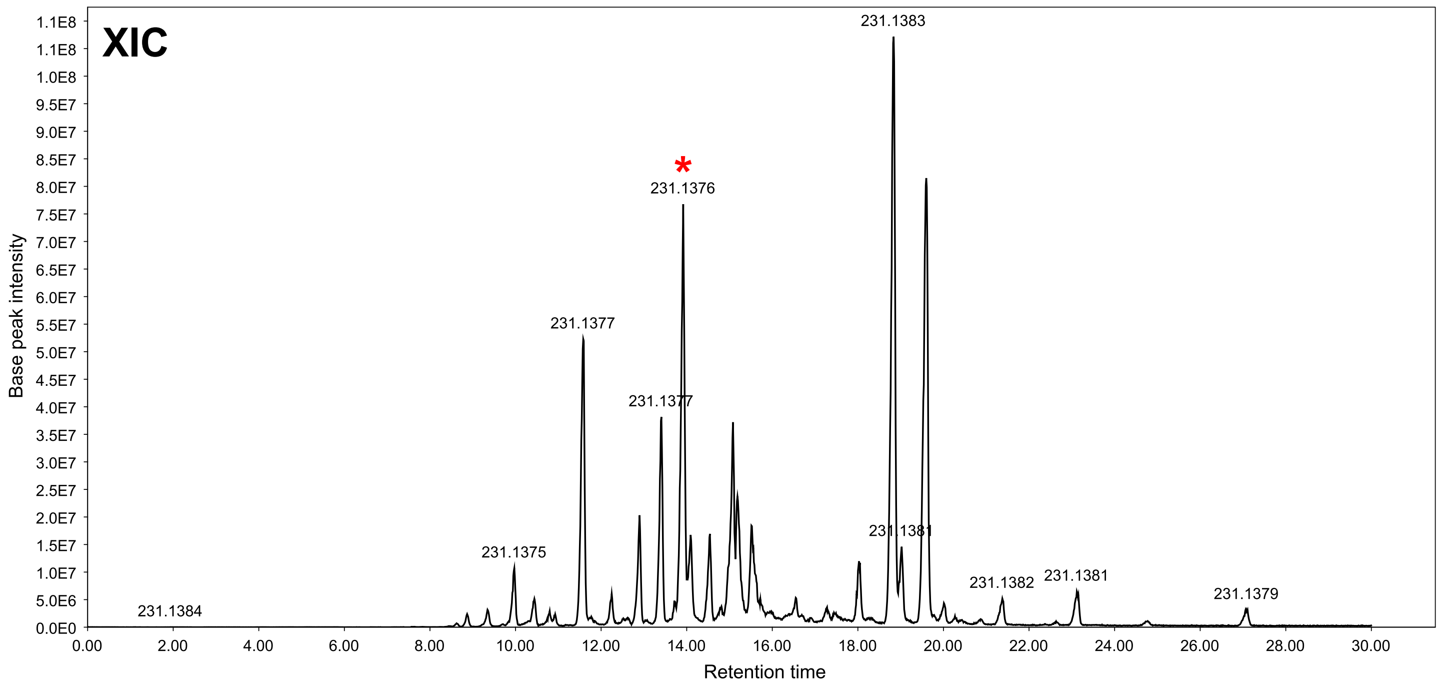

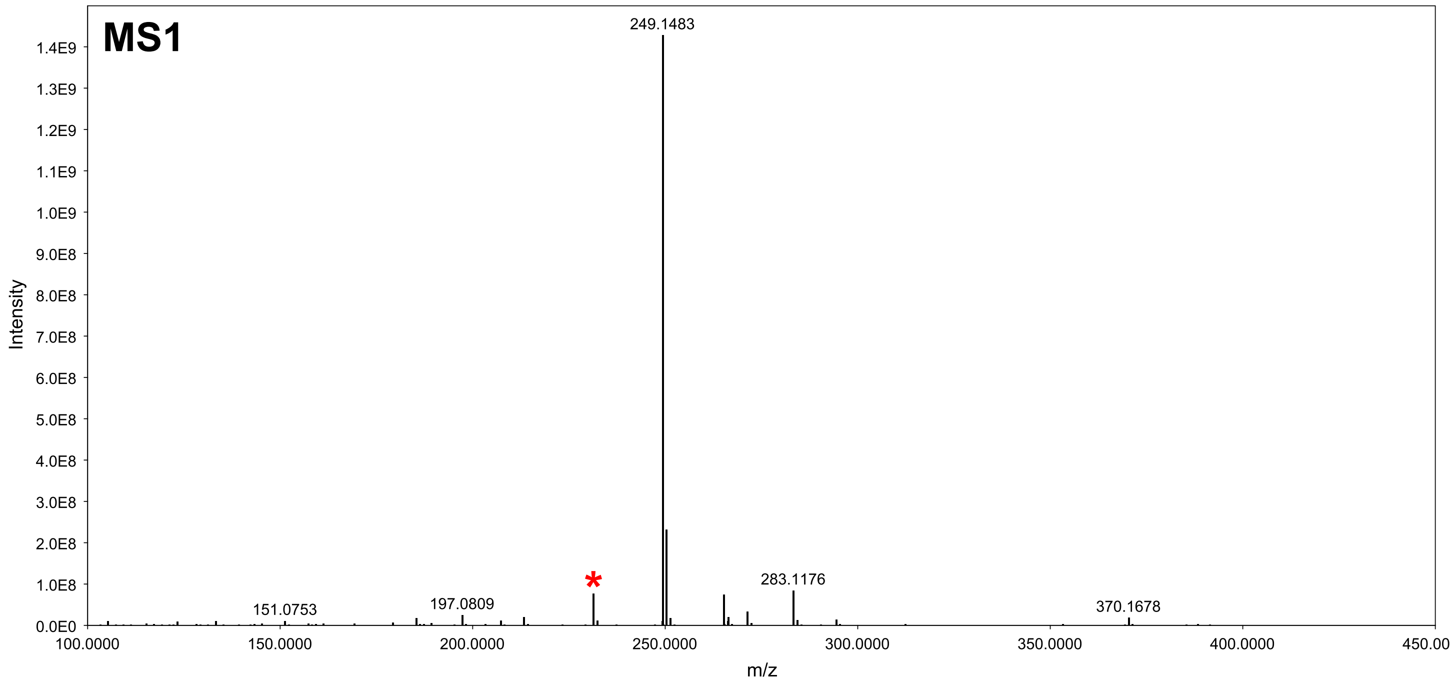

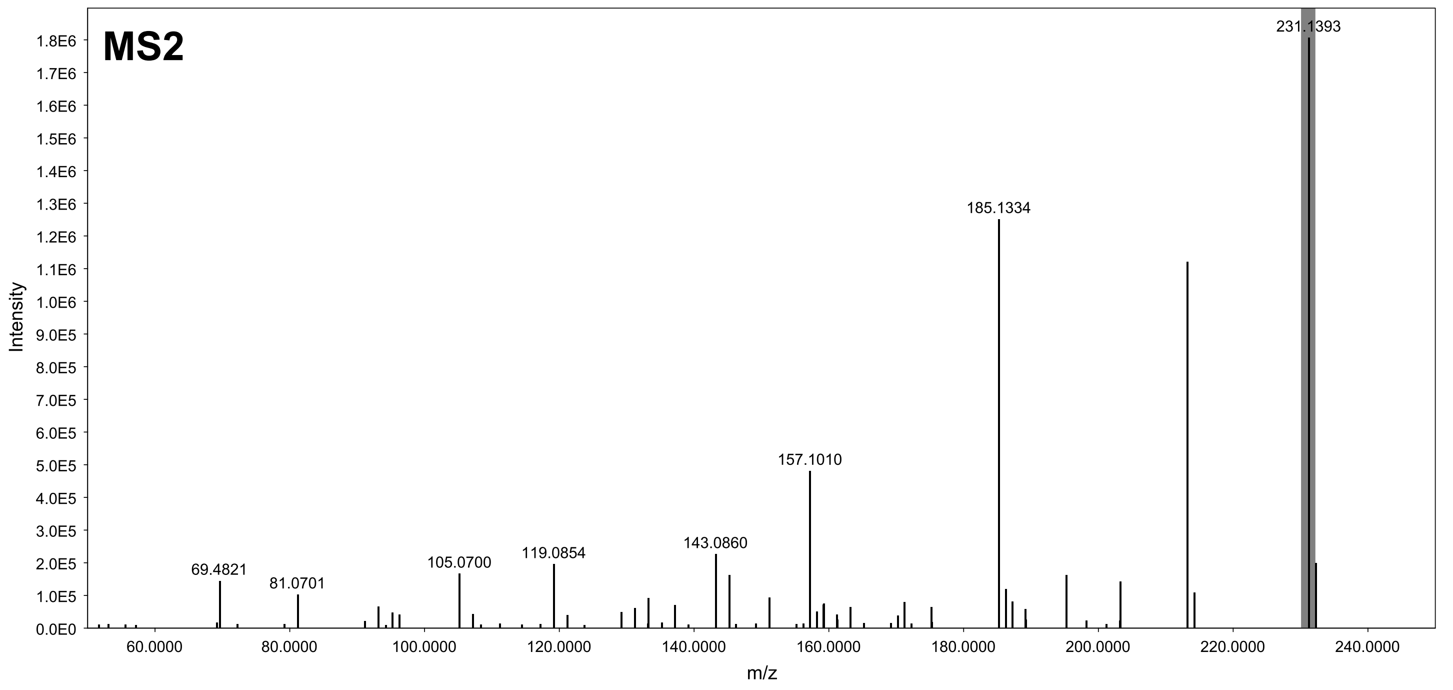

GNPS hit 12b (PDA) - XIC range m/z 231.137-231.139 [M+H]

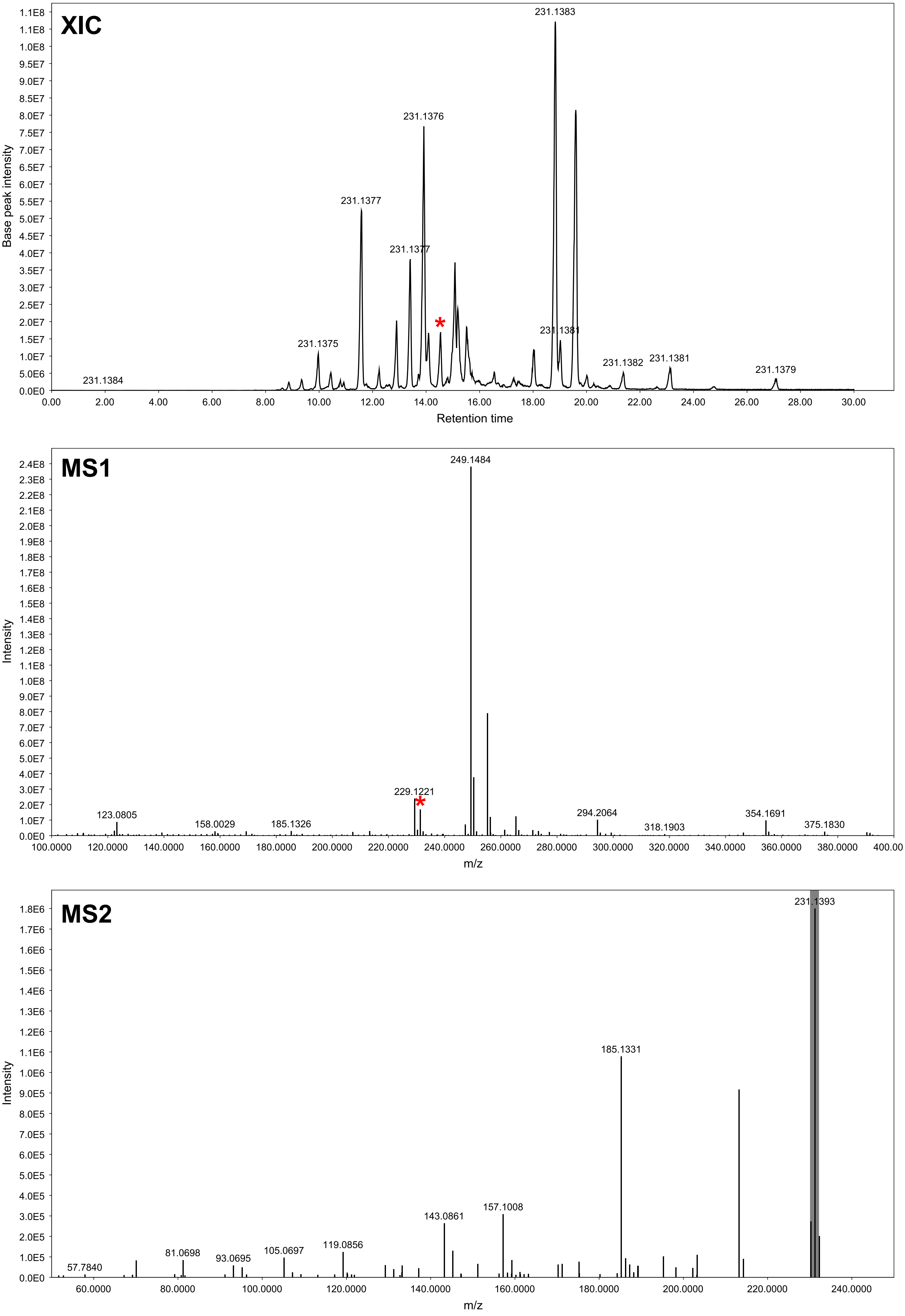

GNPS hit 12c (MEB) - XIC range m/z 231.137-231.139 [M+H]

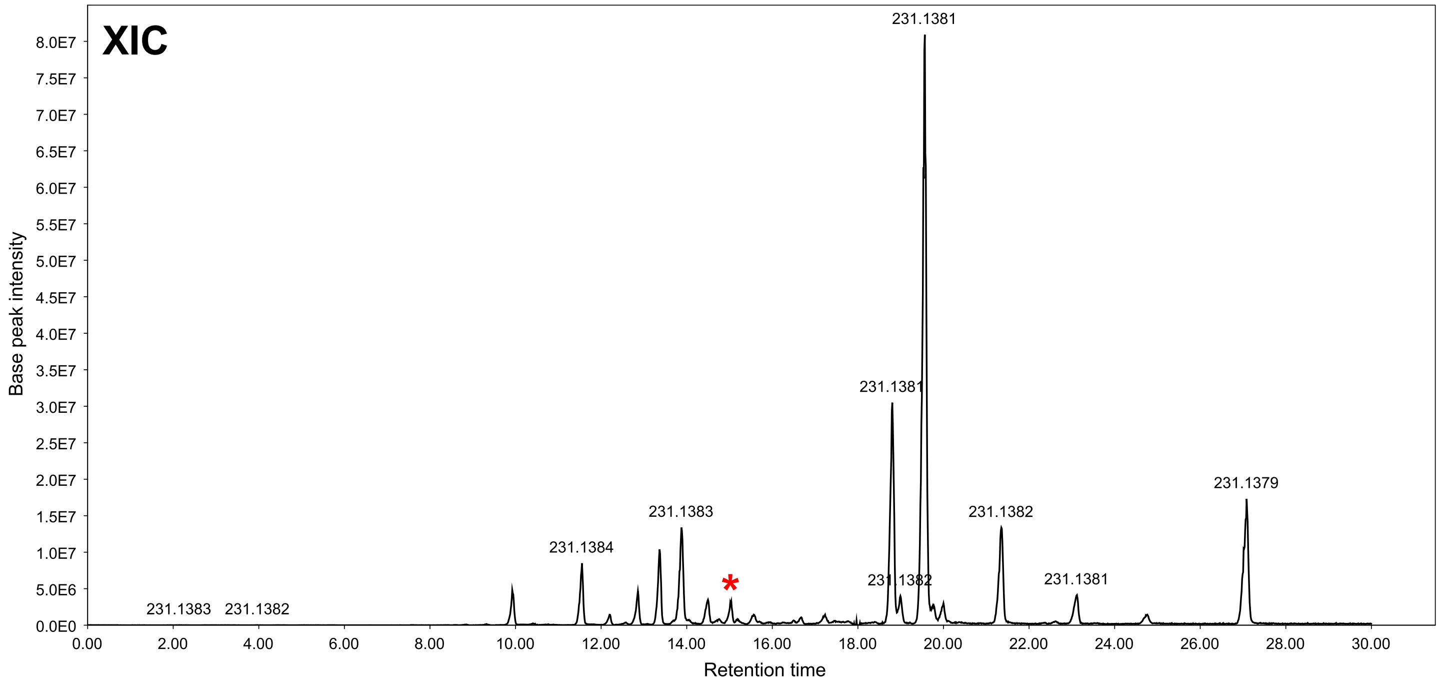

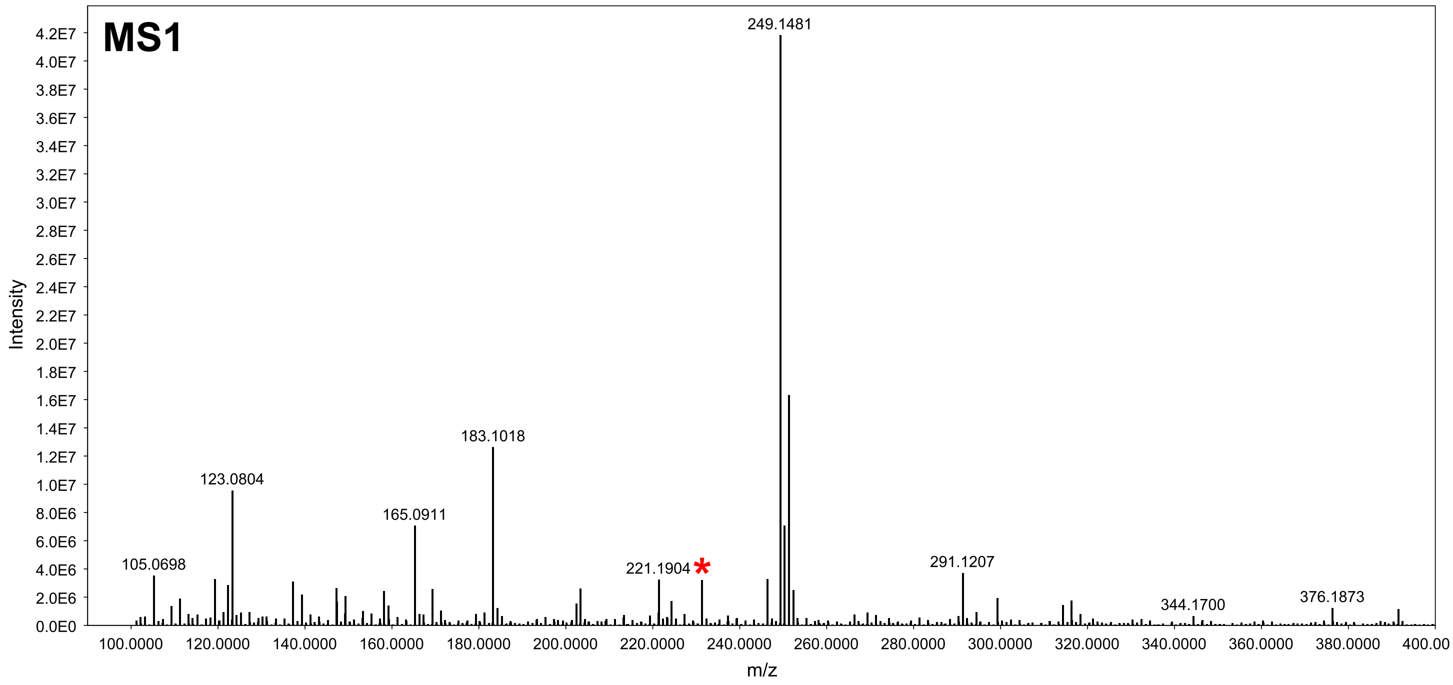

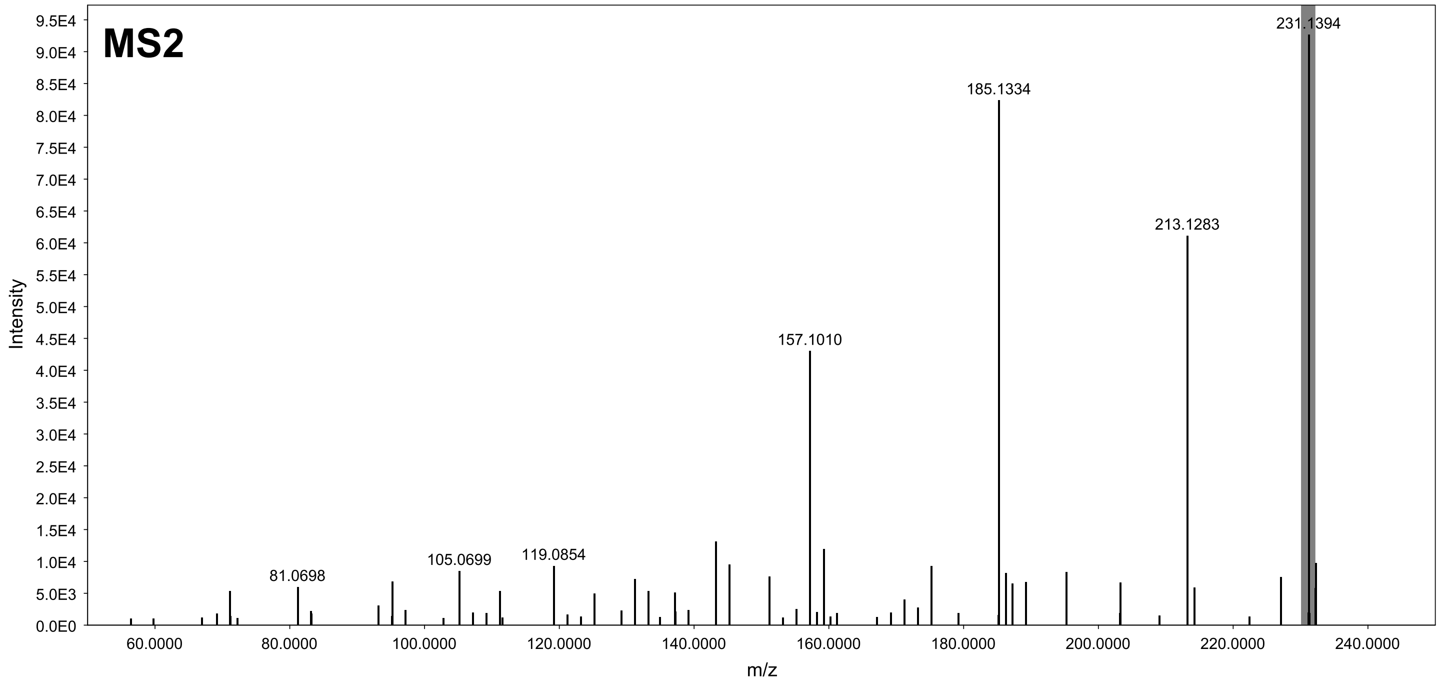

GNPS hit 13 (MEB) - XIC range m/z 607.107-607.109 [M+H]

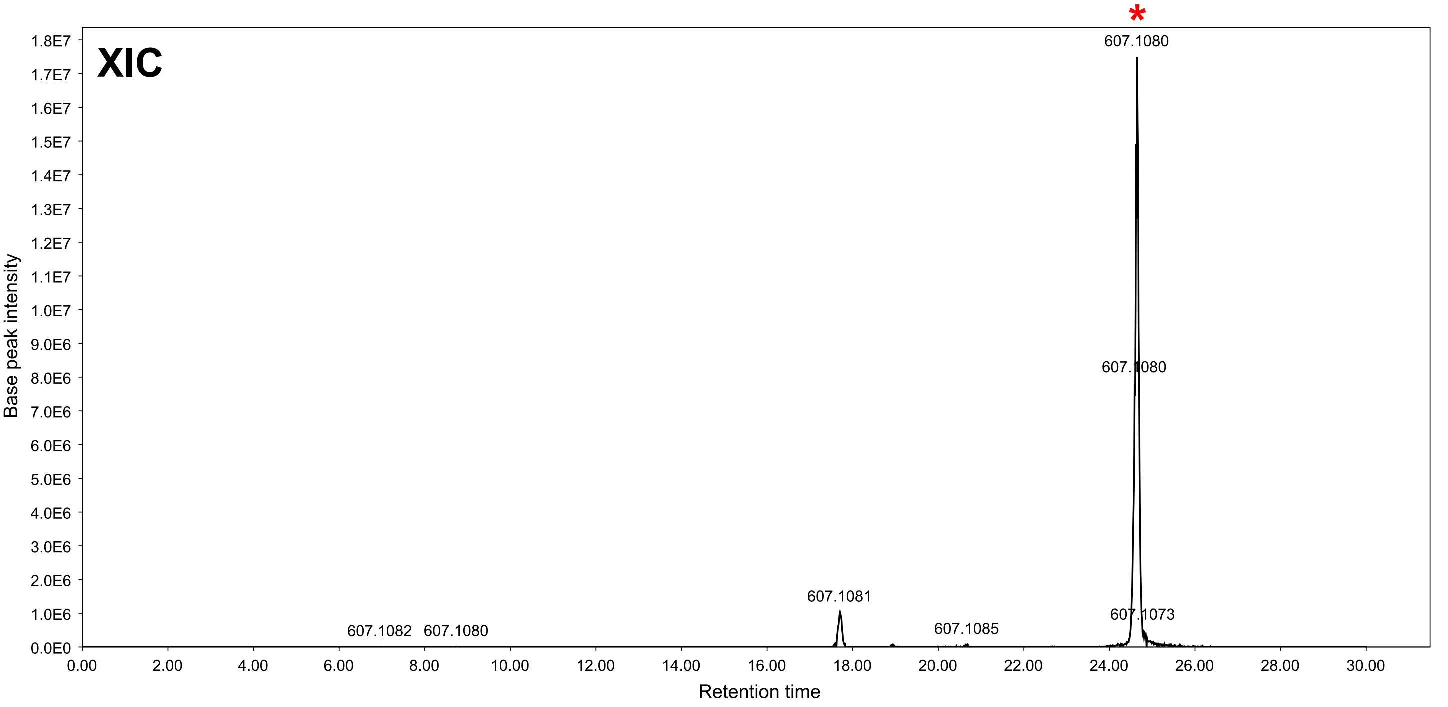

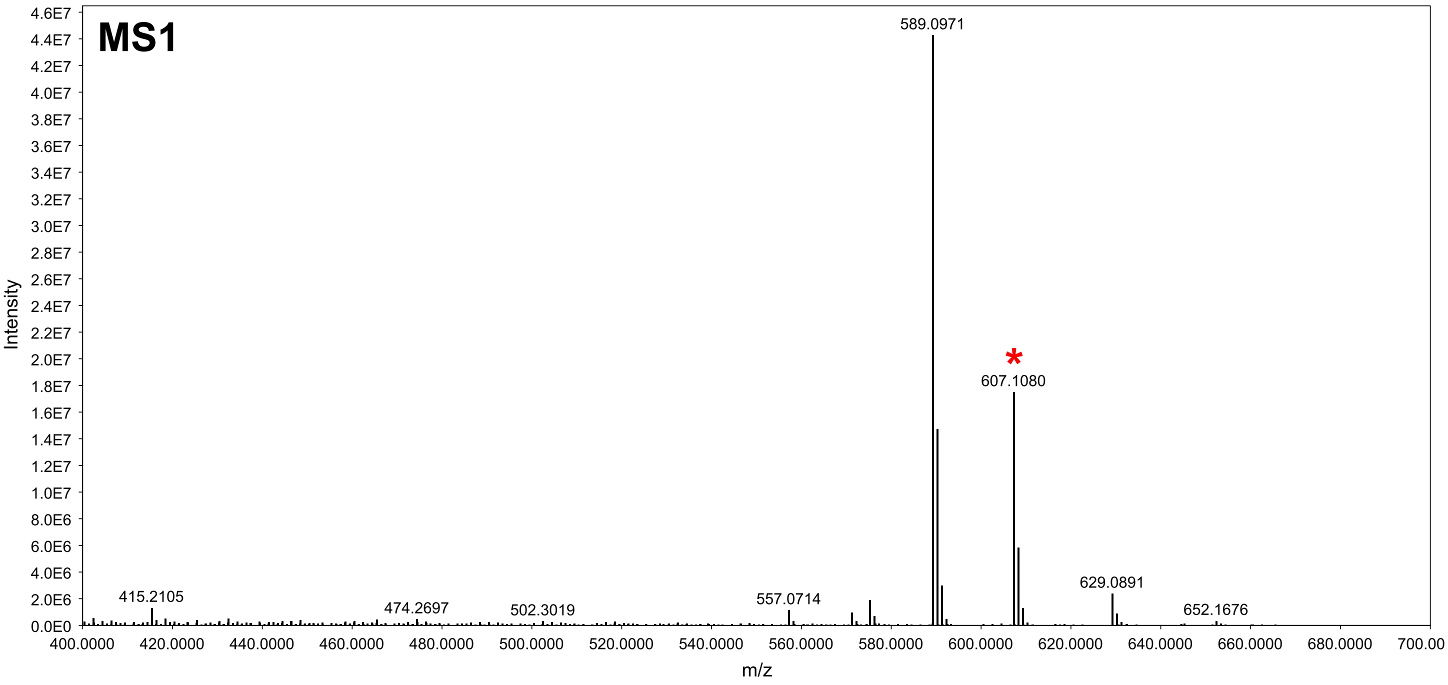

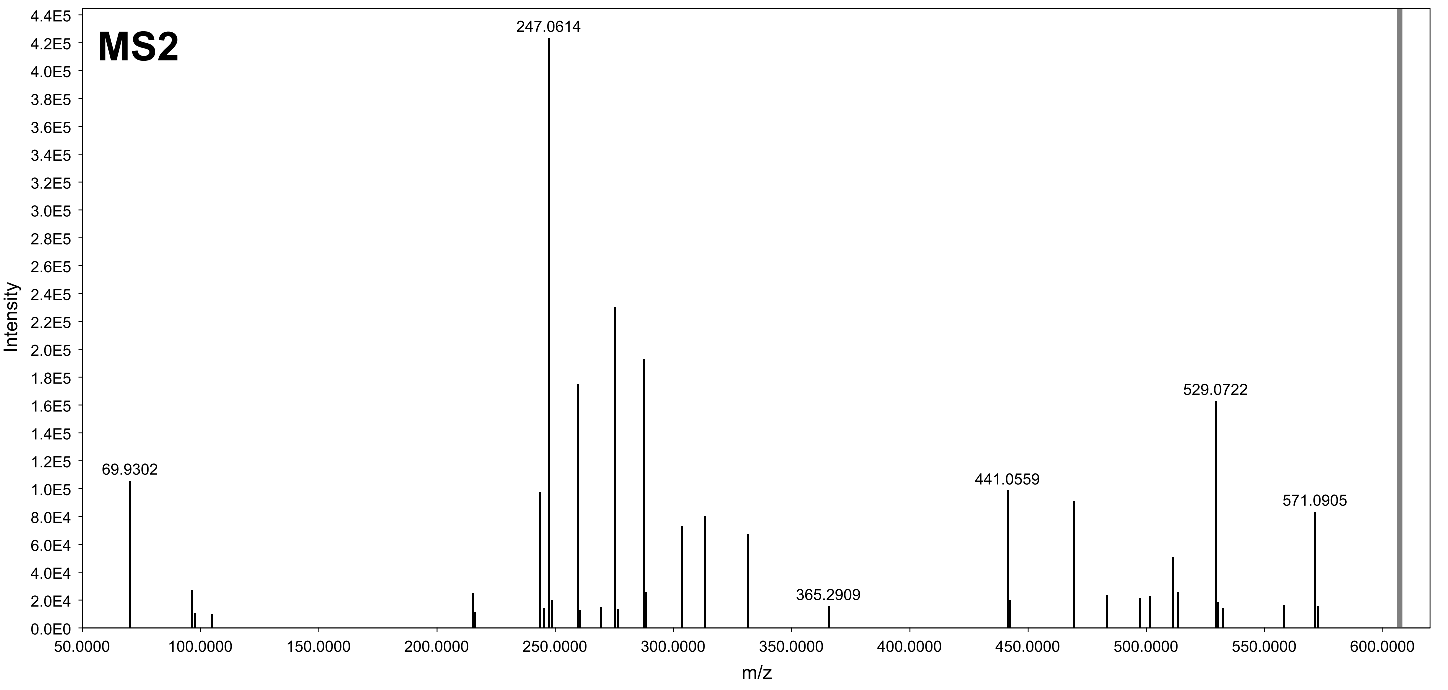

GNPS hit 14 (MEB) - XIC range m/z 267.158-267.160 [M+H]

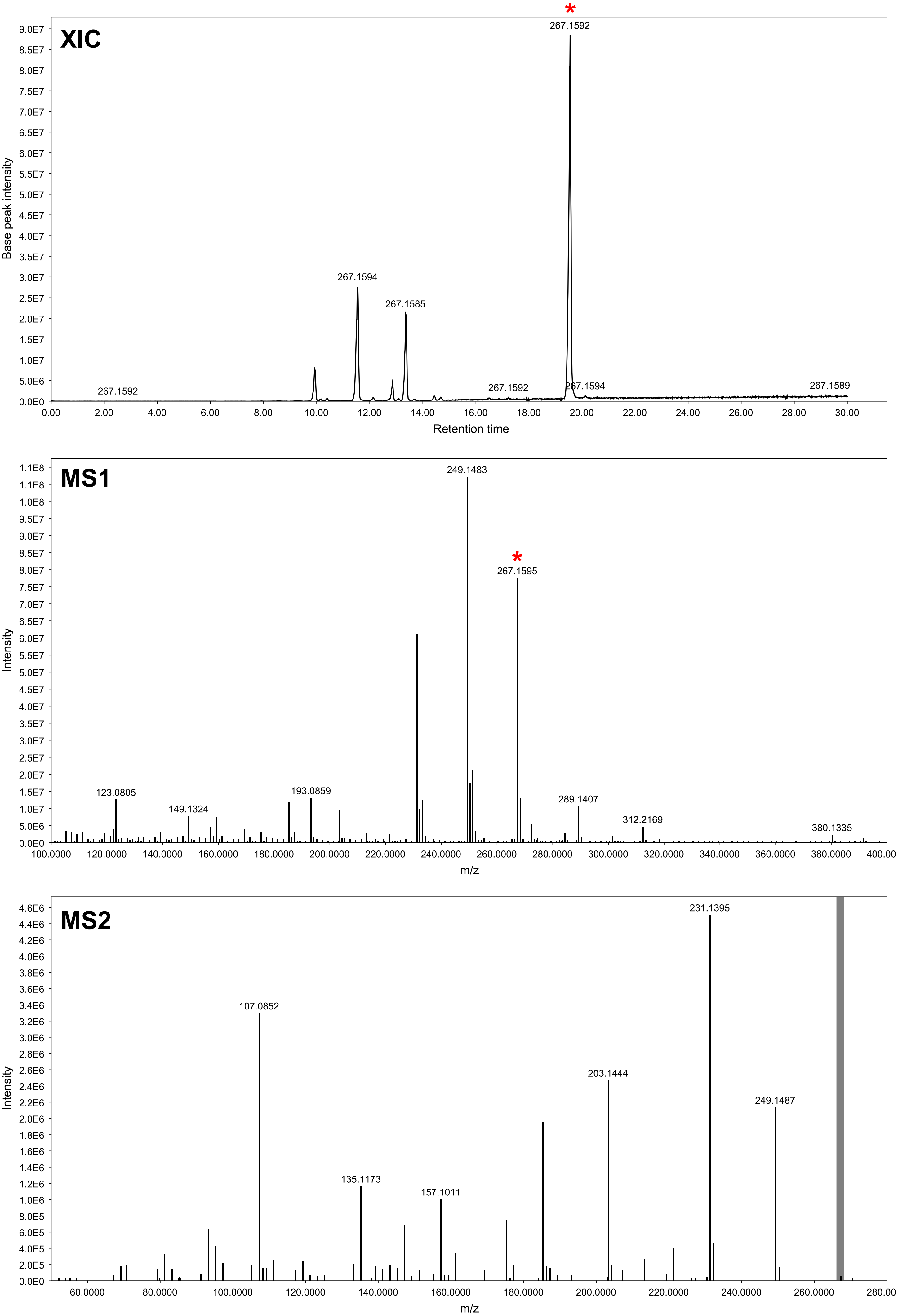

GNPS hit 15 (MEB) - XIC range m/z 233.152-233.154 [M+H]

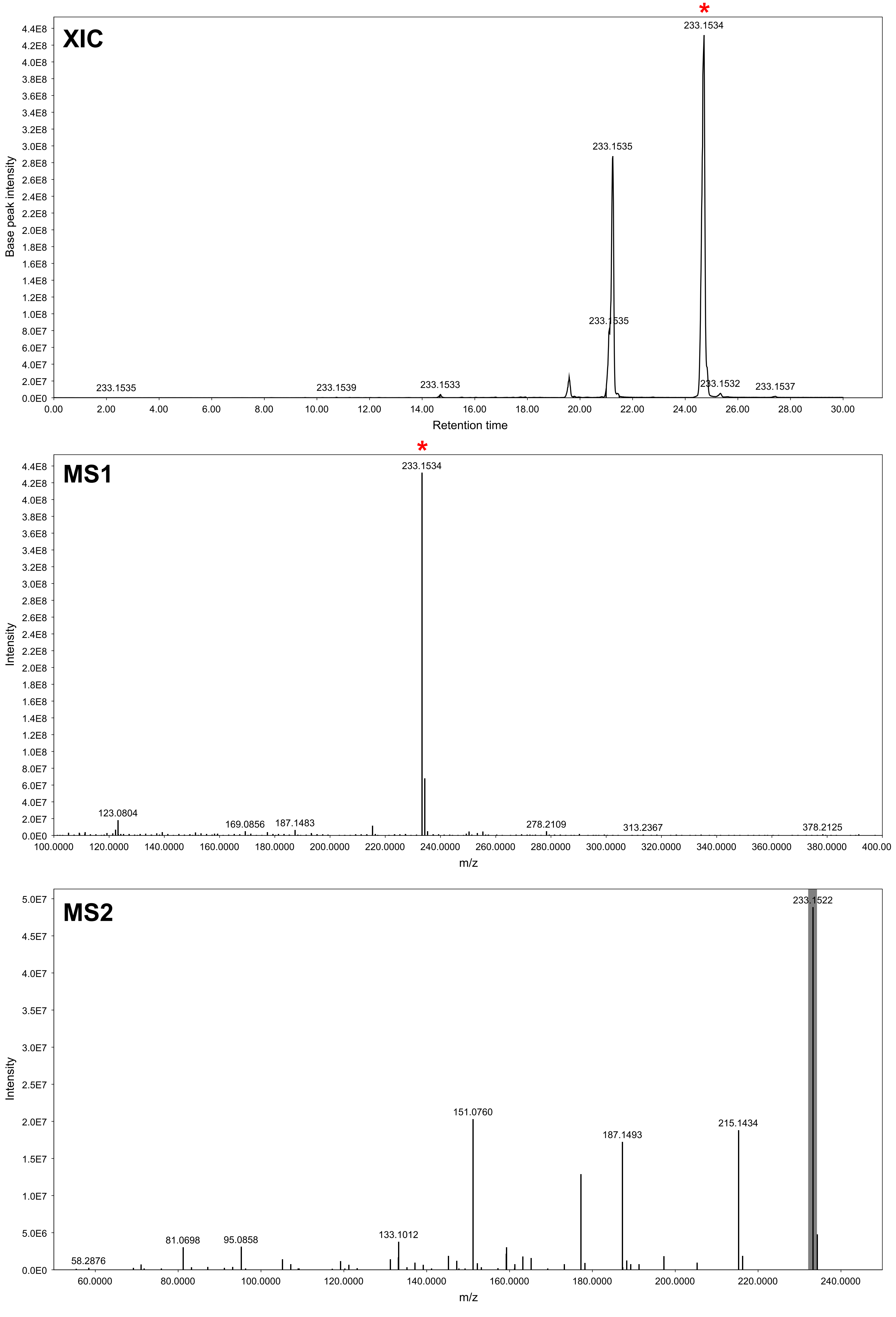

Supplement: Supplementary file 7 — Additional file 7: Extracted ion chromatograms, MS and MS/MS spectra of annotated nodes from the molecular network. [file 40694_2023_170_MOESM7_ESM.pdf]
